# Mandarin Chinese modality exclusivity norms

**DOI:** 10.1371/journal.pone.0211336

**Published:** 2019-02-20

**Authors:** I-Hsuan Chen, Qingqing Zhao, Yunfei Long, Qin Lu, Chu-Ren Huang

**Affiliations:** 1 Department of Chinese and Bilingual Studies, The Hong Kong Polytechnic University, Kowloon, Hong Kong; 2 Institute of Linguistics, Chinese Academy of Social Sciences, Beijing, China; 3 Department of Computing, The Hong Kong Polytechnic University, Kowloon, Hong Kong; 4 NIHR Nottingham Biomedical Research Centre Nottingham, University of Nottingham, United Kingdom; The University of Memphis, UNITED STATES

## Abstract

Modality exclusivity norms have been developed in different languages for research on the relationship between perceptual and conceptual systems. This paper sets up the first modality exclusivity norms for Chinese, a Sino-Tibetan language with semantics as its orthographically relevant level. The norms are collected through two studies based on Chinese sensory words. The experimental designs take into consideration the morpho-lexical and orthographic structures of Chinese. Study 1 provides a set of norms for Mandarin Chinese single-morpheme words in mean ratings of the extent to which a word is experienced through the five sense modalities. The degrees of modality exclusivity are also provided. The collected norms are further analyzed to examine how sub-lexical orthographic representations of sense modalities in Chinese characters affect speakers’ interpretation of the sensory words. In particular, we found higher modality exclusivity rating for the sense modality explicitly represented by a semantic radical component, as well as higher auditory dominant modality rating for characters with transparent phonetic symbol components. Study 2 presents the mean ratings and modality exclusivity of coordinate disyllabic compounds involving multiple sense modalities. These studies open new perspectives in the study of modality exclusivity. First, links between modality exclusivity and writing systems have been established which has strengthened previous accounts of the influence of orthography in the processing of visual information in reading. Second, a new set of modality exclusivity norms of compounds is proposed to show the competition of influence on modality exclusivity from different linguistic factors and potentially allow such norms to be linked to studies on synesthesia and semantic transparency.

## Introduction

The modality exclusivity effect is based on the observation that words referring to strong sensory meanings link to the perceptual regions of the brain [1, 2, 3, 4, 5]. Studies have shown that conceptualization corresponds to the neural systems in charge of perception and motor control [2, 6, 7]. In particular, modality exclusively mapping has been found in many studies [8, 9]; for example, studies have found that one word maps to one modality [7, 8, 10, 11]. The mapping asserts that there is a strong association between a specific word and a specific modality. However, empirical evidence has shown that a property referred to by a word may not only be conceptualized in one specific modality; instead, a property can be perceived in unimodal, bimodal, or multimodal experiences [12, 13, 14, 15, 16]. For example, English *sweet* generally refers to gustatory experience, while it can also be used to describe visual experiences as in *sweet smile*. This linguistic phenomenon is often referred to as linguistic synesthesia and generated important literature in linguistics focusing on the potentially universal directionality constraints among different senses [17, 18, 19]. The modality exclusivity norms have been developed in this context for different languages such as English and Dutch for relevant research in this field [14, 15, 16]. For instance, the modality exclusivity norms supported Strik Lievers and Winter’s [20] new study from the linguistic perspective showing that sense modalities provide cognitive motivation for grammatical categories of sensory lexicon. Since previous studies primarily focus on Indo-European languages, modality exclusivity norms for a non-Indo-European language such as Mandarin Chinese will provide an important dataset to show similarities across languages and also language-dependent variation in modality exclusivity, especially in light of recent studies showing cross-lingual variations of synesthetic mapping directionality based on Mandarin Chinese and Korean [19, 21].

The Mandarin Chinese dataset will also allow us to study the hitherto unexplored issue of the role of orthography in modality exclusivity. Sproat [22] shows that writing systems of different languages typically follow general rules of a particular linguistic module, called the orthographically-relevant-level (ORL). He also demonstrated that phonology is the ORL in most of the commonly known languages, including all languages with existing modality exclusivity data. However, Sproat [22] suggests and Huang [23] and Huang and Hsieh [24] show that semantics is the ORL for Mandarin Chinese. Taking advantage of the semantic relevance of Chinese orthography, Hung and Tzeng [25] and Tzeng and Wang [26] postulate that how people process visual information in reading is affected by their different orthographic systems. They show that subjects with phonology ORL languages are not affected by contradicting visual cues (i.e. size, color), but Chinese subjects are. Similarly, Saito, Logie, Morita, and Law [27], with a different experiment on Japanese, showed visual similarity effects but no phonological similarity effect when auditory modality is suppressed (e.g. no silent reading). Given this ORL effect on processing visual information, would orthography, especially words with explicit semantic representation of information on sense modalities, also affect processing of modal information hence influence modality exclusivity results? Note that the standard modality exclusivity studies present words in written instead of audial forms. This works well for languages with phonology ORL as the subjects are not directly affected by visual cues when they read. However, this also makes it difficult to test whether transparently encoded modality meaning would influence modality exclusivity or not. On the other hand, the attested visual effect on reading by speakers with Chinese style orthography suggests that visual orthographic information may play a role in modality exclusivity norms for these speakers.

We propose to leverage the unique features of the Mandarin writing system to test the role of orthography in modality encoding. Frist, phonological awareness [28] is well attested in reading Chinese characters [29, 30]. Second, while a Chinese character typically offers visualized information of the semantics encoded by its radical system, many characters also have a phonetic symbol that loosely represents the phonological class of the character [31]. Note that traditionally Chinese characters are classified into six types following the principles of their formation. According to the authoritative dictionary 說文解字 *Shuo Wen Jie Zi* [32], the six types are: pictographs, ideographs, compound ideographs, phono-semantic compounds, phonetic loan characters, and derived cognates. Among them, phono-semantic compounds constitute the vast majority of Chinese characters. Also note that *Shuo Wen Jie Zi* assumed that regardless of formation type, each character must contain a semantic radical. The 540 radicals provide the structure of *Shuo Wen Jie Zi* and are argued to represent core conceptual properties of the whole characters and hence group characters according to a set of basic concepts [33]. On the other hand, it is important to note that although the phonetic component similarly represents the phonological class of a character, the actual pronunciations can vary idiosyncratically and certainly do across different Chinese dialects. The assumption that these ‘phonetic symbols’ are rigorous representation of phonological identity which led to a wide ranges of changes and variations among Chinese dialects and different historical periods were important clues in the reconstruction of the phonology of Archaic Chinese and Ancient Chinese [34, 35]. This potential duality of semantic and phonological components is the feature of Chinese orthography that we will explore in this study.

Sense modalities, typically represented by the sense organs, are in fact among the basic radical groups. For example, the character 啞 *ya3* ‘hoarse, dumb’ has the radical 口 *kou3* ‘mouth’, which is a pictograph specifying the organ of making sounds, while the other component 亞 *ya3* represents the phonological class. The character 甜*tian2* ‘sweet’ is composed of two different representations of the tongue. It contains a pictographic radical 甘 *gan1*, which is the image of having something in the middle of the tongue, thus indicating the property of being sweet. The 舌 *she2* ‘tongue’ radical on the right depicts the shape of the tongue and generally is treated as representing gustatory senses. However, this component can be argued to play the additional role of a phonetic part as characters with 舌 *she2* share near identical phonological realizations, such as 舔 *tian3* ‘to lick’, and 恬 *tian2* ‘fast asleep’. As can be predicted by their functions, the characters containing the 舌 *she2* ‘tongue’ radical are originally related primarily to the gustatory modality, while the characters containing the radical 口 *kou3* ‘mouth’ could be related to gustatory modality (of eating) or auditory modality (of speaking). Some characters do not contain an identifiable radical component but still have visual information related to modality, such as the character 凹 *ao1* ‘concave’ is a pictograph of the image of a concave shape. As mentioned, many characters also have a phonetic symbol, which in fact represents the phonological group that the original pronunciation of the character belongs to but is often an opaque representation of its current pronunciation. For instance, both the radical and phonetic parts of 聾 *long2* ‘deaf’ are transparent because the character is composed of the radical 耳 *er3* ‘ear’ for auditory sense, and the phonetic 龍 *long2* ‘dragon’ for it pronunciation. However, the following three characters sharing the same phonetic part 艮 *gen3* but have very distinct pronunciations in Modern Mandarin which would make it a challenge for laypersons to identify their phonological class: 眼 *yan3* ‘eye’, 很 *hen3* ‘very’, 跟gen1 ‘to follow, with’. Given that the visual representation of phonological similarity has no effect on reading while visual cues of meanings do, we can hypothesize that it is the explicit representation of semantic knowledge that contributes to the processing effects. We can then test the sense modality effects of the transparent semantic radicals and phonetic radicals based on this hypothesis. Our expectation is the presence of a radical representing that modality will strengthen modality exclusivity for that particular sense modality. However, presence of a phonetic symbol, at least when the phonetic symbol is transparent, will strengthen the auditory modality due to the explicit activation of the auditory modality. It should be noted that not all Mandarin characters in the wordlist of sensory words have explicit associations with specific modalities. The characters differ in the degree to which their etymology is traceable. Due to the special status of Chinese characters, we will examine the influence of different types of characters on Mandarin native speakers’ judgments on sensory words based on the modality exclusivity norms collected in this paper.

In this study of Chinese modality exclusivity norms, in addition to the two orthographic components as discussed above, we will also take the morpho-lexical structure of Chinese into consideration in the experimental design. Note that in Chinese each character corresponds to a syllable and that monosyllabic and disyllabic words constitute the majority of Chinese words [36]. Hence, we focus on monosyllabic and disyllabic words separately as they are the basis for the formation of all longer words. In Study 1, we follow Lynott and Connell [14] to measure the extent to which each monosyllabic word would be perceived via visual, auditory, haptic, gustatory, and olfactory modalities, and also measure the modality exclusivity per word. We then analyze the result in terms of different compositional types of characters, in order to explore the potential contribution of Chinese radicals to the rating of modality exclusivity. In Study 2, we focus on disyllabic compound words as they are productive in Chinese word formation. In particular, a coordinate disyllabic compound may contain two characters representing different sense modalities which allows us to see the competition between two sense modalities represented by radicals at different positions in a disyllabic compound.

## Study 1: Modality ratings for single-character words

### Method

#### Participants

The participants were recruited through two crowdsourcing platforms, Sojump (https://www.sojump.com/) and Crowdflower (https://www.crowdflower.com/, superseded now by Figure-Eight https://www.figure-eight.com/). They are native speakers of Mandarin in Taiwan and Mainland China. Prior human subject ethical approval was sought and given by the Hong Kong Polytechnic University. Participants all gave their consent.

#### Materials

In Mandarin, monosyllabic and disyllabic words are the two dominant classes of lexical items but they differ in term of type and token frequency [36]. Based on Sinica Corpus [37], monosyllabic and disyllabic words together account for more than 90% of all word tokens in terms of token distribution. Each of the two categories is over 45% of total words in the corpus. Regarding word types, more than 46% of word types are disyllabic words, whereas the number of monosyllabic words is relatively restricted by the total number of available characters. Monosyllabic words dominate in terms of frequency ranks, taking up more than 90% of the most frequent 100 words. That is, regarding frequency of word tokens, monosyllabic and disyllabic words are both dominant, but there are far more disyllabic word types regarding frequency of word types. On the other hand, monosyllabic words are more dominant in terms of frequency and salience among basic words. As expected, the majority of Mandarin sensory words are either monosyllabic or disyllabic.

Each Chinese character typically represents a monosyllabic sound-meaning pair. Exceptions involve a small number of disyllabic morphemes that are represented by two characters each with a distinct syllable but without a distinct meaning. Hence the most common Mandarin compounds are disyllabic words written with two characters, and disyllabic words are typically compounds. Study 1 focuses on monosyllabic words in order to evaluate how native Mandarin speakers rate each monosyllabic word and to avoid effects from morphological complexity in rating. The wordlist is collected from Sinica Corpus [37], a 10 million word fully Part-of-Speech (PoS) tagged balanced corpus of modern Mandarin Chinese (http://asbc.iis.sinica.edu.tw/). The words included in the list belong to the adjective category. The predominant function of Mandarin adjectives is to serve as a prenominal modifier or as the head of a predicate [37]. The category of adjectives can be automatically extracted from the PoS-tagged corpus. We manually pick up monosyllabic words which can pertain to one or multiple sensory modalities from the corpus.

The Sinica Corpus contains natural occurring data, which may include typos and one-off novel usages. In order to exclude these types of tokens, the list only contains monosyllabic words which occur more than once in the corpus. As some Mandarin monosyllabic morphemes can be duplicated for intensification and aspectual modification [31, 38], we include these tokens of occurrences of monosyllabic words. Bound morphemes are excluded. Based on this criteria, there are in total 171 Mandarin monosyllabic words in the list. Each item on the wordlist is evaluated in five sensory experiences (i.e. visual, haptic, gustatory, olfactory, and auditory experiences); hence there are 855 stimuli in total.

#### Procedure

Our approach is slightly different from Lynott & Connell’s [14] modality norming approach partly due to the use of crowdsourcing platform. Lynott & Connell [14] had participants rate all 5 modalities for a word on a single screen for 423 items. For us, there are a total of 870 questions, including stimuli and golden items in our first study. When running a pilot with 10 participants based on Lynott & Connell’s [14] approach, the participants reflected that it was hard to concentrate throughout the whole list, particularly at the end. Therefore, we kept a smaller number of questions in each questionnaire for participants to stay focused through the task. In our approach, we randomly divided the questions into the 15 sets of questionnaires. Each questionnaire contains 50–60 questions as shown in S1 File. In our design, the word-modality pairs were presented separately. The complete questionnaires are available at https://osf.io/kaz78/. Each set of questionnaires has both traditional and simplified versions. The traditional character version was used in Taiwan, while the simplified character one was distributed in Mainland China. We received a total of 1,321 returned questionnaires. The numbers of responses for the total 870 questions are 29,940 from the traditional surveys and 24,141 from the simplified surveys after elimination of answers with poor quality by criteria to be introduced later in this section. The average number of responses per question is 34.41 for the traditional version, and 27.75 for the simplified version.

All the question occur in the following form adopted from Lynott & Connell [14] and Lynott and Connell [15]: 多大程度上, 您認為 (**目標詞)** 可以用來形容(**視覺, 聽覺, 觸覺, 味覺, 嗅覺)** *Duo1 da4 chen2gdu4 shang4*, *ni3 ren4wei2 (mu4biao1 ci2) ke3yi3 yong4lai2 xing2rong2 (shi4jue2*, *ting1jue2*, *chu4jue2*, *wei4jue2*, *xiu4jue2)* ‘To what extent does (the target) describe (visual, auditory, tactile, gustatory, or olfactory) experiences?’ The participants are given a scale of 0 (not at all) to 5 (greatly) to choose from. The participants were asked to rate the extent to which they experienced the modality in question based their own judgments and were informed that there were no right or wrong answers. They were also instructed that they should skip questions if they did not understand the meaning of the words. The self-paced survey took around 30 mins.

In order to verify the validity of the survey, we included baseline questions and gold-standard items. Before the survey began, we showed one high-frequency character such as 爸 *ba4* ‘father’ and 母 *mu3* ‘mother’, and then asked participants what the tone was in order to make sure that the participants could read characters. To ensure participants were native Mandarin speakers, we also included test questions such as 小明吃了蘋果跟香蕉, 請問他吃了幾種水果? *Xiao3ming2 chi1 le ping2guo3 gen1 xiang1jiao1*, *qing3wen4 ta1 chi1 le ji3zhong3 shui3guo3* ‘Xiaoming ate an apple and a banana. How many kinds of fruits did he eat?’. We excluded the answers which failed any of the test questions. To ensure the quality of the survey via crowdsourcing, we followed the procedure proposed by Caselli and Huang [39] to include a few questions with known gold standards in the experiment. We eliminated the answers which did not meet the threshold requirement of the gold standards. For example, if the word 紅 *hong2* ‘red’ is rated below 1 on the 0–5 scale for visual experiences, it does not pass the threshold. Any survey which failed two gold-standard item questions was excluded.

### Results

Based on the collected data, a dominant modality (visual, auditory, tactile, gustatory, or olfactory) was assigned to each word according to the modality which received the strongest rating. The means and standard deviations for each of the five modalities and for each dominant perceptual modality are summarized in Table 1. The details for each word are available in S2 File. In Mandarin, the greatest number of words was dominated by vision and the smallest number by olfaction. The distribution is similar to the results done in the English adjectives [14] and Dutch adjective [16], where the visual modality has the most words. The distribution of ratings for each dominant modality and the average modality exclusivity for monosyllabic words dominant in each perceptual modality are provided in Table 2. The modality exclusivity is calculated by dividing the range of ratings of the sum [14]. The modality exclusivity scores range from 10.7% to 100%; the average modality exclusivity score is 46.4%. The lowest modality exclusivity score of 0% reflects a fully multimodal concept, whereas the highest modality exclusivity score of 100% reflects an entirely unimodal concept. For instance, 暗 *an4* ‘dark’ has the exclusivity score of 94%, being fairly exclusively experienced with vision, while 微微 *wei2wei2* ‘slight’ with a modality exclusivity score of 11% is highly multimodal.

**Table 1:** Mean strength ratings (0–5) for the monosyllabic words, 95% confidence intervals, and standard deviations per perceptual modality and per dominant perceptual modality.

per Perceptual Modality			per Dominant Perceptual Modality		
Modality	Mean/CI	SD	Modality	Mean/CI	SD
Visual	3.13±0.22	1.50	Visual	4.01±0.10	0.50
Haptic	2.03±0.27	1.83	Haptic	4.40±0.15	0.51
Auditory	1.09±0.19	1.26	Auditory	3.99±0.31	0.57
Olfactory	0.77±0.18	1.20	Olfactory	4.15±0.67	0.77
Gustatory	1.34±0.25	1.66	Gustatory	4.47±0.18	0.39

**Table 2:** Mean strength rating (0–5) on the five modalities, modality exclusivity scores, and number of the monosyllabic words per dominant modality.

Dominant Modality	Strength					Modality Exclusivity	N
	Visual	Haptic	Auditory	Olfactory	Gustatory		
Visual	4.01	1.36	0.73	0.28	0.45	54%	91
Haptic	2.70	4.40	1.20	0.63	1.73	35%	43
Auditory	1.79	0.85	3.99	0.59	0.70	47%	13
Olfactory	1.15	0.38	0.51	4.15	3.6	38%	5
Gustatory	1.31	1.11	0.65	2.65	4.47	37%	19

**Fig 1:**
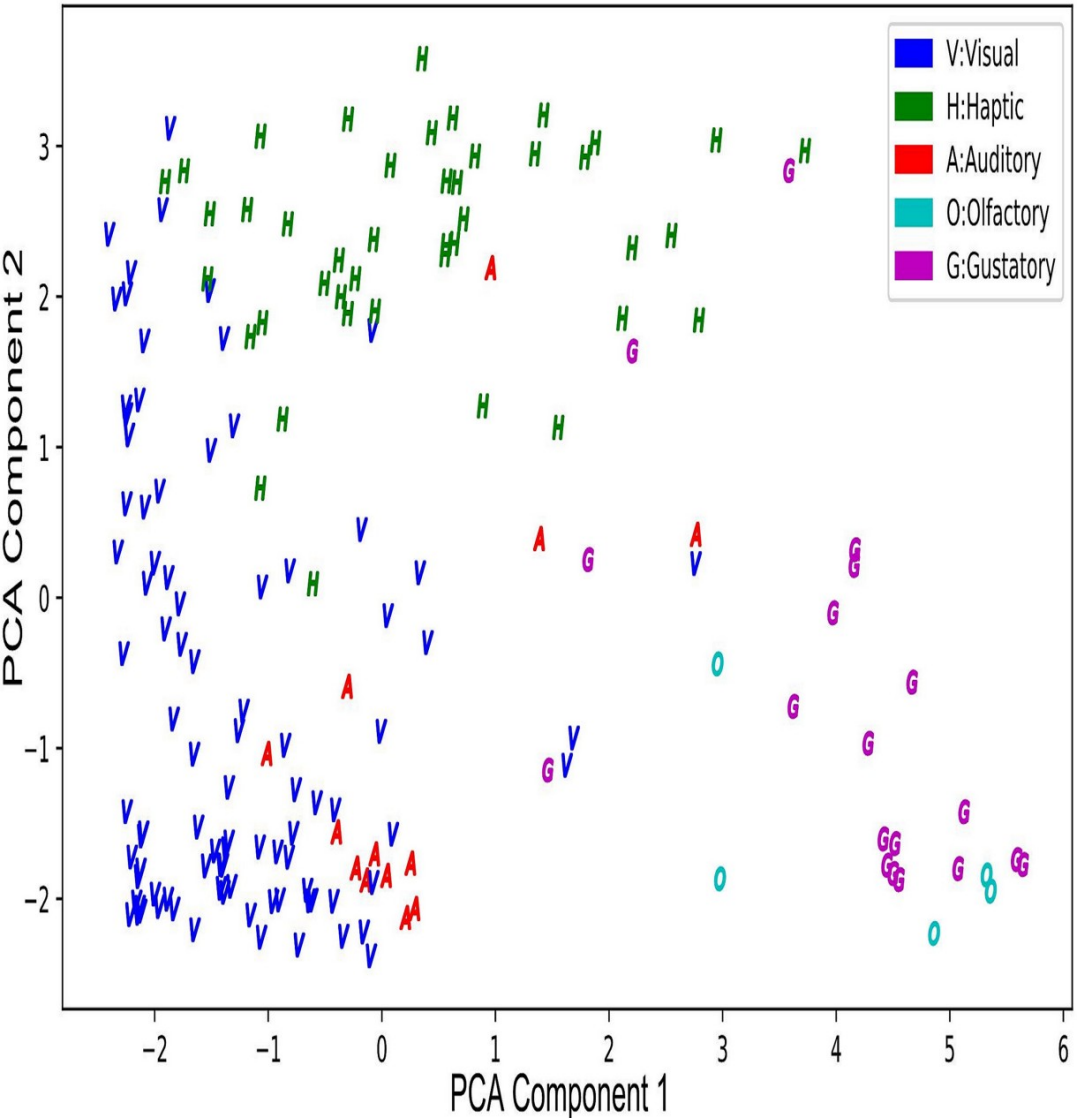
Relationship between clusters of monosyllabic words by dominant modality with relative strength on the five modalities reduced to two dimensions by principal component analysis.

The correlations between each modality are provided in Table 3. The ratings of olfactory and gustatory are strongly correlated due to their relations to food and flavors. Visual rating is negatively correlated with olfactory, gustatory, and auditory ratings. Olfactory and haptic ratings are negatively correlated. The other correlations are positive with a different degree in strength. The clustering for each modality is shown in the scatterplot in Fig, where the ratings of the five modalities are reduced to two factors by principal components analysis (PCA). The Scikit-learn Package is used for PCA, which is available in https://osf.io/kaz78/. All variables are normalized to 0–1 scale. The components are ranked based on possible variance under the constraint that a component is orthogonal to the preceding oneFig shows the first two components. The first component, constituting 39.9% of all variance, has strong positive loadings from olfactory and gustatory ratings and also has strong negative loadings from visual ratings with weak negative loadings from auditory ratings. The second component, taking up 30.3% of the variance, has strong positive loadings from haptic ratings, strong negative loadings from olfactory and gustatory modalities, and weak positive loadings from the visual and auditory modalities. The two components together explain 70.2% of the variance of the data. The clustering in Fig 1 suggests that the olfactory and gustatory modalities have a positive relationship. In addition, the visual and haptic modalities have a positive relationship particularly on the first component. As discussed in [40], human experiences of manipulating objects usually combine visual and haptic modalities. The two associations have been observed in different languages [14, 16]. Among the modalities, the auditory ratings do not have clear interactions with the other modalities, which is in line with the findings in [15]. The scores of ratings showed that the information associated with modalities is encoded in different clusters of words as observed in [41].

**Table 3:** Correlation matrix for mean strength ratings on the five perceptual modalities in Mandarin.

			Modality		
Modality	Visual	Haptic	Auditory	Olfactory	Gustatory
Visual	1				
Haptic	0.099	1			
Auditory	-0.095	0.101	1		
Olfactory	-0.386	-0.128	0.014	1	
Gustatory	-0.448	0.094	0.003	0.780	1

### Generalizations based on the norms

The data are collected from two groups: from simplified character Mandarin speakers (group A) and from traditional character Mandarin speakers (group B). In order to check whether there are regional differences, we use two-sample Z-test on the two groups of responses. The modality exclusivity is the dependent variable for the test. The result (z = 1.11, two-tailed critical value for α = 0.05 is z = 1.96) suggests that the two groups are not significantly different. The correlation of the two groups is 0.89, which also shows their similarity. In order to capture the major difference between the two groups, we also use Kullback–Leibler divergence (KL) for all the words on the list. The detailed results of each word and the KL gap between the two groups for each word are available in S3 File and https://osf.io/kaz78/. The KL results show that there is no significant difference between each modality group, but individual items have differences (p-value 0.49). The KL gap between traditional characters and simplified ones in the five modalities are shown in Table 4. For those words which have salient differences, the differences are related to how they are used in popular expressions in different Mandarin-speaking regions. Based on how a word has been evaluated in each modality, the words have the biggest differences and the smallest differences are also summarized in Table 4. In the group with the most salient differences, 蔫 *nian2* ‘withering’ is not a frequent word in colloquial conversations. The participants thus differ in judgment in multiple modalities. 正 *zheng4* ‘upright’ can be used to describe superb gustatory experiences in recent years in Taiwan, but not in other Mandarin-speaking areas. The new meaning of 正 *zheng4* ‘upright’ may influence the participants’ evaluation. Similarly, 潮 *chao2* ‘damp’ and 癟 *bie3* ‘shrunken’ appear in newly coined expressions, but the new meanings are popular only in one region and have not widely spread to all Mandarin-speaking regions yet. The emergence of new meanings accounts for the regional differences in the rating task. In the group with the smallest difference, the KL test shows that the responses are very consistent across traditional character and simplified character speakers.

**Table 4:** Differences between traditional and simplified character users: The KL gap per modality and the KL gaps for words with the biggest and smallest difference.

Traditional-simplified KL gap		Most different words			Least different words		
Modality	KL Gap	Word	Modality	KL Gap	Word	Modality	KL Gap
Visual	0.48	蔫 *nian2* ‘withering’	Haptic	1.03	滿 *man3* ‘full’	Visual	0
Haptic	0.48	蔫*nian2* ‘withering’	Visual	1.03	淺 *qian3* ‘shallow’	Auditory	0
Gustatory	0.36	正 *zheng4* ‘upright’	Gustatory	0.89	壯 *zhuang4* ‘strong’	Gustatory	0
Olfactory	0.30	潮 *chao2* ‘damp’	Haptic	0.85	蒼蒼 *chang1-chang1* ‘hazy’	Haptic	0
Auditory	0.41	癟 *bie3* ‘shrunken’	Haptic	0.66	纖纖 *xian1-xian1* ‘slender’	Olfactory	0

Although the wordlists may vary in different studies for modalities, similarities among the languages can be observed. The ratings of Mandarin monosyllabic adjectives reflect multimodal concepts, which is line with the results of the English adjectives studied by Lynott and Connell [14]. The visual dominance [14, 15, 16, 42, 43] is also observed in the present study. Regarding the modality with the least exclusivity, it is not consistent across languages. In Mandarin, haptic modality appears to be the least dominant one in modality exclusivity. Gustation is found to be the least dominant modality for English adjectives in [15] and in Dutch [16].

### Further analysis: Orthographical influences on evaluating modalities

Based on the modality exclusivity norms of single characters words, we further analyze the distribution of different character types and their relations with the five modalities. Our aim is to identify potential correlations between orthography with semantic ORL and modality exclusivity based on the previous findings that speakers of these language incorporates visual information when reading. The etymology of Chinese characters is shown in their components: pictographic, ideographic and/or radicals and phonetic symbol [24, 44]. Some of the radicals explicitly specify the connections with a certain modality. For example, in Study 1 甜*tian2* ‘sweet’, which contains two pictographic radicals derived from the shape of tongue (舌 *she2* ‘tongue’ and 甘 *gan2* ‘taste good’), is rated to be primarily associated with gustation. The character 吵 *chao3* ‘noisy’, which has a high score of exclusivity in the auditory modality, has the radical 口 *kou3* ‘mouth’. The examples suggest the connection between the radicals and the perception of modalities. We posit, based on the orthography-motivated visual-cue triggered reading strategy of Chinese speakers, that the visual cue of a radical explicitly representing a sense modality will favor that particular modality in exclusivity ratings.

Based on the authoritative dictionary 說文解字 *Shuo Wen Jie Zi* [32], Chinese characters can be classified into six types according to their formation rules: pictographs, ideographs, compound ideographs, phono-semantic compounds, phonetic loan characters, and derived cognates. It is well-known that phono-semantic compounds constitute the vast majority of Chinese characters; hence most of the characters in our wordlist belong to this category. We classified characters of the words included in the norms according to how sense modality related information is represented by their orthographic component(s): pictographs, ideographs, compound ideographs, and phono-semantic compounds. Of the six classes of characters, phonetic loan characters and derived cognates are not included in the analysis because none of the words in the list falls into the two categories.

Pictographs, which are considered to be the most original type of word formation, represent the visual image of the object. For instance, 目 *mu4* ‘eye’ is the image of an eye. Ideographs are based on iconicity, which are used to describe abstract ideas, as in 一 *yi1* ‘one’ and二 *er4* ‘two’. Ideographs may also contain iconic modification of pictographs. Compounds composed of more than two ideographs or pictographs are ideographic compounds. For example, 森 *sen1* ‘forest/ dense’ is a compound of three pictographs 木*mu4* ‘tree’, indicating the density of trees. Phono-semantic compounds are composed of two components: a semantic component from pictographs or ideographs and a phonetic symbol. For instance, 瀟 *xiao1* ‘whistling’ has the water radical 氵 with the phonetic symbol, 蕭 *xiao1*, which imitates the whistling sound from nature. Based on *Shuo Wen Jie Zi* [32], all the characters on the list were classified into the four categories for analysis.

The percentages of the character types in the five sensory modalities are shown in Table 5. It should be noted that most of the words belong to the phono-semantic type since this is the most predominant class of Chinese characters according to all previous studies. Ideographic compounds are found in the five modalities, while pictographs are found in the visual, gustatory, and haptic modalities. Ideographs are found only in visual and gustatory modalities.

**Table 5:** Six types of characters in the five dominant modalities.

	Visual Dominant Modality					Gustatory Dominant Modality				
		Modality Exclusivity			SR		Modality Exclusivity			SR
Type	Proportion	Weak	Moderate	Strong	Mean	Proportion	Weak	Moderate	Strong	Mean
Phono-semantic compound	66%	9.8%	45.9%	44.2%	3.82	63%	33%	11%	58%	4.21
Ideographic compound	20%	5.6%	33.3%	**61.1%**	**4.13**	26%	40%	40%	20%	4.91
Pictograph	13%	8.3%	58.3%	33.3%	**4.66**	5%	0%	100%	0%	5
Ideograph	1%	0%	100%	0%	3.59	5%	0%	0%	100%	5
	Olfactory Dominant Modality					Auditory Dominant Modality				
		Modality Exclusivity			SR		Modality Exclusivity			SR
Type	Proportion	Weak	Moderate	Strong	Mean	Proportion	Weak	Moderate	Strong	Mean
Phono-semantic compound	80%	25%	75%	0%	3.98	85%	27.3%	18.2%	**54.5%**	**4.13**
Ideographic compound	20%	0%	100%	0%	4.84	15%	50%	50%	0%	3.25
	Haptic Dominant Modality					(SR: strength rating based on 0–5 scale)				
		Modality Exclusivity			SR					
Type	Proportion	Weak	Moderate	Strong	Mean					
Phono-semantic compound	77%	35%	53%	12%	4.37					
Ideographic compound	18%	50%	50%	0%	4.15					
Pictograph	13%	50%	50%	0%	4.56					

Pictographs represent visual images. Ideographic compounds include pictographic components. These two image-based types of characters provide salient visual input. In the visual modality, pictographs have the highest rating score and ideographic compounds have the second highest rating score. In the wordlist, the ideographic compounds have the strongest modality exclusivity. In order to compare phono-semantic characters and image-based characters in terms of the rating scores, we used the Mann-Whitney U test to show the difference. The results show that the distribution of phono-semantic compounds is significantly different from both ideographic compounds (U = 468.5, n_1_ = 18, n_2_ = 60, p < 0.2, two-tailed) and pictographs (U = 188, n_1_ = 12, n_2_ = 60, p < 0.05, two-tailed). Notably, phono-semantic compounds also provide some visual information since their semantic components can be pictographs or ideographs. However, the visual information is not as direct as that of pictographs and ideographic compounds. Although the phono-semantic characters take up the majority of Chinese characters, the relation between image-based characters and visual modality can still be observed.

The phonetic symbol of a phono-semantic compound represents a phonological class for the original pronunciation of the character. Recall that in past studies [27] showing no phonological similarity effects and lack of sensory meaning per se of the phonetic symbols, we do not expect a direct influence of them on modality exclusivity. However, on the other hand, as the phonetic symbol represents a phonological class and requires activation of phonological knowledge (i.e. classification of auditory input), it is conceivable that it could have effect in that modality. And in fact, among words in the auditory dominant modality, phono-semantic compounds receive the highest rating score. Particularly, 54.5% of the phono-semantic compounds are strong in modality exclusivity.

In the gustatory dominant modality, the ideographic and pictographic characters contain the mouth-shape radical, 口 *kou3* ‘mouth’. The radical specifies the important organ for gustatory experiences. Due to the relation, they received the highest ratings. Similarly, in the olfactory and haptic modalities, the ideographic compounds and pictographs have image-based radicals referring to the olfactory and haptic modalities respectively. The two types of characters also have high rating scores.

Although the proportion of types of characters is not balanced, the influence of the types of characters is reflected in the scores of ratings and modality exclusivity. The image-based types of characters, in particular, may have higher rating scores and stronger modality exclusivity in the modality which is indicated by the components of the characters.

## Study 2: Modality rating for two-character compounds

In Mandarin, a disyllabic sensory word can be composed of two monosyllabic sensory words from the same sensory experiences or from different sensory experiences. These two-character sensory words are often used to describe the sensory experiences related to the etymology of its components. For example, the disyllabic word 濕潤 *shi1run4* ‘moist’ is composed of two characters with similar meanings: ‘wet’ and ‘moist’ respectively. The two characters are both from the haptic modality, and the compound still describes haptic experiences. These disyllabic words can also have two component characters from different sense modalities. For example, 痛苦 *tong4ku3* ‘painful’ is composed of two characters from haptic experiences (‘pain’) and gustatory experiences (‘bitterness’) respectively. However, the meaning of this disyllabic word is basically haptic. The term then was extended to the mental interpretation of suffering.

Study 2 focuses on a special type of disyllabic words, which are different from the previously introduced compositional compounds. This type of disyllabic words is formed by two characters from two different sensory modalities, yet the word can describe experiences from a third modality. For example, in 濃烈 *nong2lie4* ‘intense’, the first character is associated with gustation and the second one with the haptic modality (in the sense of heat of fire). However, the word can also be used to describe olfactory experiences. In order to examine how the Mandarin native speakers evaluate this type of complex disyllabic words, we did a rating task on modalities in Study 2.

### Method

#### Participants

Thirty-five participants (average age 33.5) were recruited through the crowdsourcing platforms Sojump. They are native speakers of Mandarin in Taiwan (19 participants: female 12, male 7) and Mainland China (16 participants: female 6, male 7).

#### Materials

Any disyllabic adjective describes sense modality experiences in a modality different from the modalities of its two components is included in the current study. A total of sixty-one words are collected. The modality associated with the character components is based on their etymological origins according to 說文解字 *Shuo Wen Jie Zi* [32] and 說文解字注 *Shuo Wen Jie Zi Zhu* ‘Notes for explaining graphs and analyzing characters’ [45] via the online version 漢典 *Handian* (http://www.zdic.net/). For the characters not included in the two dictionaries, the definition is based on 漢語大字典 *Hanyu Dazidian* ‘Chinese big dictionary’ [46]. The wordlist has 10 out of 20 possible combinations: Auditory-Gustatory, Auditory-Haptic, Gustatory-Haptic, Gustatory-Visual, Haptic-Auditory, Haptic-Gustatory, Haptic-Olfactory, Haptic-Visual, Visual-Gustatory, and Visual-Haptic. Since a combination of involving olfactory only appears once, 7 of the 10 gaps can be easily explained as due to the small number of olfactory words. The other three gaps involve auditory words (Auditory-Visual, Gustatory-Auditory, and Visual Auditory). This may be partly due to that auditory sensory words are most likely to have a non-nominal (i.e. neither noun nor adjective [20]), and hence are less likely to appear in coordinative compounds with sensory senses. It is possible that there are also other underlying cognitive motivations as Jo [21] reports similar gaps involving olfactory and auditory modality for Korean compounds. The complete set of lists is available at https://osf.io/kaz78/.

The stimuli of Study 2 appeared in the same format as that of Study 1, as shown in S4 File. The version in traditional characters was distributed in Taiwan; the version in simplified characters was distributed in Mainland China.

#### Procedure

The participants were self-selected responding to our call. They were asked to rate the extent to which they experienced of the modality in question. Before the survey began, the participants were instructed that they should make the judgments on their own. They were also told that they could skip questions if they did not understand the meaning of the word. This experiment was administered online via Sojump. The self-paced experiment took around 45–60 mins.

### Results

We excluded six answers of the received questionnaires because they failed to pass the baseline questions and gold-standard items, which are the same as those in the Study 1. Based on the rating of the disyllabic words, the means and standard deviations for each of the five modalities and for each dominant perceptual modality are summarized in Table 6. The details for each two-character compound are available in S5 File and https://osf.io/kaz78/. A dominant modality (visual, auditory, tactile, gustatory, or olfactory) is assigned to each disyllabic word based on the modality which received the strongest rating. Table 7 summarizes the ratings for each dominant modality and the average modality exclusivity for the disyllabic words dominant in each perceptual modality. According to the results, the visual modality has the most compounds, whereas the olfactory modality has the least. The distribution is similar to that of the single-character adjectives in Study 1. The visual modality has the highest modality exclusivity score in either single-character words or two-character words. Modality exclusivity scores in the list range from 6% to 67.1%, with an average of 36%. The score is lower than that of single-character words in Study 1. A comparison between Table 2 for Study 1 and Table 7 for Study 3 shows that the modality exclusivity is lower in disyllabic words in each of the five modalities. Overall, the scores of modality exclusivity show that disyllabic compounds are found to be relatively multimodal.

**Table 6:** Mean strength ratings (0–5) for the disyllabic words, 95% confidence intervals, and standard deviations per perceptual modality and per dominant perceptual modality.

per Perceptual Modality			per Dominant Perceptual Modality		
Modality	Mean/CI	SD	Modality	Mean/CI	SD
Visual	3.11±0.11	0.82	Visual	3.65±0.15	0.41
Haptic	1.82±0.36	1.44	Haptic	4.34±0.25	0.4
Auditory	2.43±0.22	0.88	Auditory	3.48±0.81	0.92
Olfactory	1.34±0.29	1.16	Olfactory	4.55±0	0
Gustatory	2.07±0.40	1.58	Gustatory	4.13±0.24	0.65

**Table 7:** Mean strength rating (0–5) on the five modalities, modality exclusivity scores, and number of the disyllabic words per dominant modality.

Dominant Modality	Strength					Modality Exclusivity	N
	Visual	Haptic	Auditory	Olfactory	Gustatory		
Visual	3.65	1.57	2.53	0.65	0.83	47%	28
Haptic	3.27	4.34	2.09	0.69	1.93	30%	10
Auditory	2.38	1.57	3.47	1.50	1.84	18%	5
Olfactory	2.00	0.56	0.89	4.56	4.15	33%	1
Gustatory	2.39	0.92	1.82	2.63	4.13	27%	17

The average of the ratings in each modality for the 10 types of compounds is summarized in Table 8, where the highest score is bolded and the second one is underscored. For the majority of types in Table 8, the most dominant modality is the same as one of the compound components, such as Gustatory-Visual, Gustatory-Haptic, Auditory-Haptic, and Auditory-Gustatory types. If the most dominant modality is not the same as one of the components, at least the secondary dominant modality is the same as one of the components. For example, the Haptic-Auditory type has the dominant modality in visual modality and the secondary one in auditory modality. However, not all the compounds have the dominant modality from one of their components. For example, the Haptic-Gustatory and Haptic-Olfactory types have vision as the most dominant modality and the auditory modality as the secondary one. The most dominant modality and the secondary one are associated with neither of the components. The results show that the meaning of the compounds is not strictly compositional and that the concatenation of two modalities in compounding cannot exclude the possibility of a third modality being the dominant one. However, even with the introducing of a new dominant modality, some correlations can be found between the compound composition and the dominant modality. For example, we found that compounds in the visual dominant modality typically contain at least one character representing the haptic modality. For compounds with the haptic dominant modality, the component characters are mainly associated with either visual or haptic modality. In addition, Haptic-Gustatory and Haptic-Olfactory compounds end up having the visual modality as the dominant one. This implies a strong possibility of mapping from haptic to vidual modalities. And interestingly, for compounds in the group with the dominant auditory modality, none of their component characters are associated with the auditory modality. Instead, these compounds typically belong to the Visual-Haptic type. Again, since the auditory sense is shown to be the most frequent target of synesthetic mapping, and both visual and haptic as the more common sources [20, 47](Strik Lievers and Winter 2018, Zhao and Huang 2018), this result suggests that there could be a strong causal relation between modality exclusivity of coordinate compounds and linguistic synaesthesia.

**Table 8:** Average of ratings for each combination of compounds.

	Visual	Haptic	Auditory	Olfactory	Gustatory
G-V	2.14	0.65	1.72	2.94	**3.41**
G-H	2.10	0.60	1.73	2.49	**3.92**
A-G	3.22	0.44	3.04	2.56	**3.78**
A-H	3.63	**4.74**	2.59	0.78	1.93
H-A	**3.87**	3.09	3.83	2.41	3.04
H-G	**3.75**	1.49	2.41	1.02	1.88
H-O	**4.00**	1.19	2.59	0.52	0.78
H-V	**3.36**	2.70	2.21	0.66	1.01
V-G	2.09	1.22	1.94	2.54	**4.25**
V-H	**3.38**	1.91	2.59	0.80	1.38

The average of the ratings in each modality for the 10 types of compounds is summarized in Table 8, where the highest score is bolded and the second one is underscored. Excluding the olfactory sense compounds from comparison since for lack of contributing olfactory component in these compounds, and with synesthetic mapping directionality in mind, the pattern can be predicted. As expected, compounds with visual sense have the strongest exclusivity, and regardless of whether the compound contains a visual component or not. For gustatory sense compounds, ones with the highest exclusivity are those with a gustatory component. However, for compounds with haptic sense, the exclusivity scores are generally low, with the exception of both directions of auditory-haptic combinations. And for auditory sense compounds, although they do not have top exclusivity scores, there are no lowest ones either. And most interestingly, there are three compound patterns without auditory component but which have the second highest exclusivity score in the auditory modality: Haptic-Gustatory, Haptic-Olfactory, and Visual-Haptic. A possible explanation for this middle range exclusivity with cross-sensory near dominance is the fact that the auditory sense is commonly shown to be at the end of directionality of synesthetic mapping and hence is the most likely to be the sensory domain described by other senses in terms of both possible combinations [17], as well as frequency [18]. Zhao and Huang [47] confirmed the mapping directionality position and highest frequency in synesthesia for visual modality words in Chinese. Interestingly, olfactory sense compounds also rank relatively high in terms of exclusivity with three secondary dominant exclusivity ratings for compounds without olfactory components: Gustatory-Haptic, Gustatory-Visual, and Visual-Gustatory. Here the explanation is different as olfactory sense is typically considered to be in the middle of directionality of synesthetic mapping. These second highest exclusivity ratings most likely occur because of the close affinity between olfactory and gustatory sense words [18]. This explanation is supported by the fact that in all three compound patterns where olfactory received the second highest rating, the gustatory sense was in fact always the dominant one.

Last, but not the least, the extreme contrast of high and low exclusivity rating for compounds with haptic sense and the across-the-board high ratings for compounds with visual senses can also be accounted for when synesthesia is taken into consideration. Haptic modality is often considered at the beginning of synesthetic mapping directionality, which means that it can describe other senses but other senses rarely describe it. Hence, the modality exclusivity ranking is predicted to be low for compounds that do not have haptic components. For the visual modality, as it is both inherently dominant and a likely target of synesthesia (typically considered to be parallel to or just before the auditory modality), high exclusivity can be achieved by either maintaining the same modality of a component or by synesthesia. In sum, the result of Study 2 confirmed our suspicion that linguistic synesthesia plays an important role in the modality exclusivity rating for compound words in Chinese.

## Discussion

This rating task shows that the semantics encoded in the components of the two-character words may influence the participants’ evaluation. For the majority of the compounds, their dominant modality is still related to the etymology of the component characters. In the visual, haptic, and gustatory dominant modalities, the dominant modality is the same as one of the two components for the majority of the compounds. Notably, the compounds which are rated to be dominant in the visual modality can contain no visual component. Within this group, the compounds contain one haptic component, which strongly suggests either synesthetic or associative relations between the visual and haptic modalities.

Among the disyllabic compounds, those with the gustatory dominant modality contain components from all four other modalities, whereas those with olfactory dominant modality has the least diversity. Thus, as originally planned, our modality exclusivity rating of Mandarin compounds containing two characters with radicals representing different sense modalities yield direct evidence and potential generalizations about the compatibility and relation among the five sense modalities. Results of this rating task suggests that both morpho-lexical structure and lexical semantic operations, including linguistic synesthesia in particular, can interact with the inherent exclusivity tendencies of the five sense modalities to arrive at the exclusivity ratings of the two-character compounds. Directionality of synesthetic mapping, neuro-cognitive connectivity, and inherent differences in the linguistic encoding of different sense modalities are a few of the main potential motivations to account for the complex inter-play of component (non)-represented modalities and compound exclusivity rating.

## General discussion

This current study is designed to reflect characteristics of the morphology and orthography of Mandarin Chinese, as well as the role they may play in reading strategy. The rating tasks on the Mandarin modality exclusivity are done in both single-character and two-character compound words in this paper in order to examine how Mandarin native speakers evaluate sensory words with different morphological complexity. The exclusivity ratings of mono-syllabic words written with one character allows clarity in the identification of original and basic sense modality as well as the relationship between that modality and all highly rated modalities. The experiment on disyllabic two-character compounds provides the context for us to observe more complex interactions among multiple sense modalities. Our results show that the sense modality property of a word is generally multimodal for both mono- and di-syllabic words, which is in line with the modality ratings of other languages [14, 15, 16]. In both studies, the visual dominance is salient for both monosyllabic words and disyllabic compounds. In particular, compounds with the visual dominant modality can generally describe sensory experiences not explicitly represented by either component of the compounds.

The analysis of both semantic radical and phonetic symbols of Chinese characters shows how a writing system may influence the conceptualization of words. Characters with semantic radicals or visual cues explicitly representing a sense modality lead to higher scores in modality exclusivity rating for the relevant modality. Crucially, our studies also show that characters containing a phonetic symbol tend to have higher modality exclusivity rating scores in the auditory modality. This is an interesting result as phonological coding’s influence on the auditory modality cannot be shown in languages with writing systems with phonology as the single ORL. Furthermore, previous studies on Chinese and Japanese suggest that there are no phonological effects unless the verbal/auditory channel is involved. To account for this unexpected result, we suggest that it could be attributed to the fact that the semantic writing of Chinese plays a role in attuning speakers to process visual cues of meaning [26], especially when extra-linguistic visual senses are introduced. In other words, perhaps this generalization of processing strategy can be extended to visual cues of auditory information. Similar to the facilitation effect when a sense modality is explicitly represented, the perception of a phonetic symbol in a character facilitates the auditory modality because it explicitly encodes information about the auditory modality.

## Conclusion

The paper provides the first sets of modality exclusivity ratings of a language with explicit representations of sense modality in the writing system. Regardless of different types of component composition, Chinese characters typically have a semantic radical that contains explicit information related to the experiences from different modalities. Leveraging this encoded modality information in our analysis, the collected modality exclusivity norms show that Chinese orthography plays an important role in the perception of the modality of a word. The most interesting discovery is that the awareness of direct phonological encoding in a character seems to strengthen the exclusivity rating of the auditory modality. We also set up a set of exclusivity norms for compounds involving components encoding different modalities. We found that the dominant modality of such compounds could come from the modality of one or neither of the components. However, it is conceivable that other variables such as semantic transparency of compound and component words [48] may also play a role. In addition, expansion and elaboration of compound modality exclusivity norms in Chinese and other languages may lead to new directions of research.

Last, but not the least, the issue of cognitive motivations of grammatical categories is a crucial one both for neuro-cognitive sciences and for linguistics. Strik Lievers and Winter [20] have shown that a careful study of the sensory lexicon incorporating modality exclusivity norms may shed light on this important question. It is expected that this current study can contribute to a deeper understanding of the interaction between sense modality and sensory lexicon and add to the empirical resources needed to eventually unravel the cognitive bases of human language.

## Supporting information

S1 File Study1: Survey (traditional character version). (PDF)

S2 File Study1: Modality exclusivity norms for single-character words. (XLSX)

S3 File Study1: Differences between traditional characters and simplified characters. (XLSX)

S4 File Study2: Survey (traditional character version). (PDF)

S5 File Study2: Modality exclusivity norms for two-character compounds. (XLSX)

## References

[1] Barros-Loscertales A, Gonzalez J, Pulvermüller F, Ventura-Campos N, Bustamante J, Costumero CV, et al Reading salt activates gustatory brain regions: fMRI evidence for semantic grounding in a novel sensory modality. Cerebral Cortex. 2012;22(11):2554–2563. 10.1093/cercor/bhr324 22123940 PMC4705335

[2] Barsalou LW. Perceptual symbol systems. Behavioral and Brain Sciences. 1999;22(4):577–660. 10.1017/S0140525X99532147 11301525

[3] Goldberg RF, Perfetti CA, Schneider W. Perceptual knowledge retrieval activates sensory brain regions. Journal of Neuroscience. 2006;26:4917–4921. 10.1523/JNEUROSCI.5389-05.2006 16672666 PMC6674166

[4] Kiefer M, Sim EJ, Herrnberger B, Grothe J, Hoenig K. The sound of concepts: Four markers for a link between auditory and conceptual brain systems. The Journal of Neuroscience. 2008; 28(47): 12224–12230. 10.1523/JNEUROSCI.3579-08.2008 19020016 PMC6671691

[5] Simmons WK, Ramjee V, Beauchamp MS, McRae K, Martin A, Barsalou LW. A common neural substrate for perceiving and knowing about color. Neuropsychologia. 2007;45(12):2802–2810. 10.1016/j.neuropsychologia.2007.05.002 17575989 PMC3596878

[6] Glenberg AM, Kaschak MP. Grounding language in action. Psychonomic Bulletin & Review. 2002;9:558–565. 12412897 10.3758/bf03196313

[7] Pecher D, Zwaan RA, editors. The grounding cognition: The role of perception and action in memory, language, and thinking. Cambridge: Cambridge University Press; 2005.

[8] Newman SD, Klatzky RL, Lederman SJ, Just MA. Imagining material versus geometric properties of objects: An fMRI study. Cognitive Brain Research. 2005;23:235–246. 10.1016/j.cogbrainres.2004.10.020 15820631

[9] Marques JF. Specialization and semantic organization: Evidence for multiple semantics linked to sensory modalities. Memory & Cognition. 2006;34:60–67. 16686106 10.3758/bf03193386

[10] Popova Y. (2005). Image schemas and verbal synaesthesia In: Hampe B, Grady J, editors. From perception to meaning: Image schema in cognitive linguistics (Cognitive Linguistics Research 29). Berlin & New York: Mouton de Gruyter; 2005 p. 395–420.

[11] Shen Y. Cognitive constraints on poetic figures. Cognitive Linguistics. 1997;8(1):33–71.

[12] Connell L. Representing object colour in language comprehension. Cognition. 2007;102:476–485. 10.1016/j.cognition.2006.02.009 16616075

[13] Ernst MO, Bülthoff HH. (2004). Merging the senses into a robust percept. Trends in Cognitive Sciences. 2004;8:162–169. 10.1016/j.tics.2004.02.002 15050512

[14] Lynott D, Connell L. Modality exclusivity norms for 423 object properties. Behavior Research Methods. 2009;41(2):558–564. 10.3758/BRM.41.2.558 19363198

[15] Lynott D, Connell L. (2013). Modality exclusivity norms for 400 nouns: The relationship between perceptual experience and surface word form. Behavior Research Methods, 45(2), 516–526. 10.3758/s13428-012-0267-0 23055172

[16] Speed LJ, Majid Asifa. Dutch modality exclusivity norms: Simulating perceptual modality in space. Behavior Research Methods. 2017;49(6):2204–2218. 10.3758/s13428-017-0852-3 28155185

[17] Williams J. Synaesthetic adjectives: A possible law of semantic change. Language. 1976;52(2):461–478.

[18] Strik Lievers F. Synaesthesia: A corpus-based study of cross-modal directionality. Functions of Language. 2015;22(1):69–95.

[19] Zhao QQ, Huang CR, Long YF. Synaesthesia in Chinese: A corpus-based study of gustatory adjectives in Mandarin. Linguistics. 2018;56(5):1167–1194.

[20] Strik Lievers F, Winter B. Sensory language across lexical categories Lingua. 2018;204(1):45–61.

[21] Jo C. (2018). Synesthetic Metaphors in Korean Compound Words. In: Devereux B, Shutova E, Huang CR, editors. Proceedings of Workshop on Linguistic and Neuro-Cognitive Resources (LiNCR) at LREC 2018; 2018 May 8; Miyazaki. p. 38–44.

[22] Sproat R. A computational theory of writing systems. Cambridge: Cambridge University Press; 2000.

[23] Huang CR. Semantics as an Orthography-Relevant Level for Mandarin Chinese. In: The 17th Annual Conference of the International Association of Chinese Linguistics; 2009.

[24] Huang CR, Hsieh S. Chinese lexical semantics: from radicals to event structure In: Wang SY, Sun C, editors. The Oxford handbook of Chinese linguistics. New York: Oxford University Press; 2015 p290–305.

[25] Hung DL, Tzeng OJ. Orthographic variations and visual information processing. Psychological Bulletin. 1981;90(3): 377 7302050

[26] Tzeng OJ, Wang SY. The first two R's: The way different languages reduce speech to script affects how visual information is processed in the brain. American Scientist. 1983;71(3):238–243. 6881679

[27] Saito S, Logie R, Morita A, Law A. Visual and phonological similarity effects in verbal immediate serial recall: A test with kanji materials. Journal of Memory and Language. 2008;59(1):1–17.

[28] Mann VA. Phonological awareness: The role of reading experience. Cognition. 1986;24(1–2):65–92. 3791922 10.1016/0010-0277(86)90005-3

[29] Perfetti CA, Zhang S. Phonological processes in reading Chinese characters. Journal of Experimental Psychology: Learning, Memory, and Cognition. 1991;17(4):633–673.

[30] Tzeng OJ, Hung DL, Wang SY. Speech recoding in reading Chinese characters. Journal of Experimental Psychology: Human Learning and Memory. 1977;3(6):621.

[31] Huang CR, Shi D, editors. A Reference Grammar of Chinese. Cambridge: Cambridge University Press; 2016.

[32] Xu S. Shuo wen jie zi ‘Explaining graphs and analyzing characters’ Beijing: Zhonghua Book Company; 1963 [156].

[33] Chou YM, Huang CR. Hantology: conceptual system discovery based on orthographic convention In: Huang CR, Calzolari N, Gangemi A, Lenci A, Oltramari A, Prévot L, editors. Ontology and the Lexicon: A Natural Language Processing Perspective. Cambridge: Cambridge University Press; 2010 p. 122–143.

[34] Karlgren B. Grammata serica recensa. Bulletin of the Museum of Far Eastern Antiquities. 1957;29:1–332.

[35] Norman J. Chinese. Cambridge University Press; 1988.

[36] Huang CR, Chen C, Shen C. The nature of categorical ambiguity and its implications for language processing: a corpus-based study of Mandarin Chinese In: Nakayama M, editor. Sentence Processing in East Asian Languages Stanford. California: CSLI Publications; 2002 p. 53–83.

[37] Chen KJ, Huang CR, Chang L, Hsu H. Sinica Corpus: Design methodology for balanced corpora. Proceedings of 11th Pacific Asia Conference on Language, Information and Computation (PACLIC 11); 1996 Dec 20–22; Seoul, South Korea. p. 167–176.

[38] Huang S, Jin J, Shi D. Adjectives and adjective phrases In: Huang CR, Shi D, editors. A reference grammar of Chinese. Cambridge: Cambridge University Press; 2016 p. 276–296.

[39] Caselli T, Huang CR. Sourcing the crowd for a few good ones: event type detection. Proceedings of 24th International Conference on Computational Linguistics (COLING 2012); 2012 Dec 10–14; Mumbai .1239–1248.

[40] Louwerse M, Connell L. A Taste of Words: Linguistic Context and Perceptual Simulation Predict the Modality of Words. Cognitive Science. 2011;35:381–398. 10.1111/j.1551-6709.2010.01157.x 21429005

[41] Levinson SC, Majid A. Differential ineffability and the senses. Mind & Language. 2014;29:407–427. 10.1111/mila.12057

[42] Van Dantzig S, Cowell RA, Zeelenberg R, Pecher D. A sharp image or a sharp knife: Norms for the modality-exclusivity of 774 concept-property items. Behavior Research Methods. 2011:43(1),145–154. 10.3758/s13428-010-0038-8 21287109 PMC3048290

[43] Winter B. Taste and smell words form an affectively loaded part of the English lexicon. Language, Cognition and Neuroscience. 2016;31(8):975–988.

[44] Wang N. Xungu xue yuanli ‘The principle in etymological studies on Chinese’. Beijing: China International Radio Press; 1996.

[45] Duan Y. Shuo wen jie zi zhu ‘Commentary on explaining graphs and analyzing characters’. Nanjing: Phoenix Press; 2007.

[46] Xu Z. Hanyu da zidan ‘Great compendium of Chinese characters’ 2nd ed Chengdu & Wuhan: Sichuan Dictionary Publishing Company & Hubei Dictionary Publishing Company; 2010 [1986].

[47] Zhao QQ, Huang CR. A Study on the Mapping Model and Underlying Mechanisms of Synaesthetic Metaphors in Mandarin 现代汉语通感隐喻的映射模型与制约机制. 《语言教学与研究》Language Teaching and Linguistic Studies. 2018;189: 44–55.

[48] Wang S, Huang CR, Yao Y, Chan SA. The effect of morphological structure on semantic transparency ratings. Language and Linguistics 20.2. Forthcoming 2019.

