# Supplementary material for: Mandarin Chinese modality exclusivity norms
Source: PLoS One. 2019 Feb 20;14(2):e0211336. doi: 10.1371/journal.pone.0211336 (PMC6382104; doi:10.1371/journal.pone.0211336)
Supplement: S1 File — (PDF) [file pone.0211336.s001.pdf]

背景：选择背景 小 自定义问卷页眉 手机版外观

感覺詞標註實驗(GROUP 4/12)

很抱歉，由于此问卷长时间没有接收到答卷，问卷被停止，如有需要请 恢复运行

說明: 人類的感覺大致可以分為5種,即視覺、聽覺、觸覺、味覺和嗅覺。因為有視覺,我們可以看到五彩繽紛的世界,因為有聽覺,我們能聽到各種各樣的聲音。而因為我們擁有觸覺,我們能感受到溫度的變化、物體材質的不同,偶爾我們的身體還能感受到疼痛等。與此同時,味覺能讓我們嚐到食物的不同味道,而嗅覺能讓我們聞到世界上各種不同的氣味。
在漢語中,有一批詞可以用來形容我們的感覺。在本次的實驗中,我們想邀請您來判斷,這些詞在多大程度上可以形容我們的這五種感覺。比如說,我們將給您如下的問題「多大程度上,您認為(清)可以用來形容視覺?」您將需要在1、2、3、4、5、6這六個選項中做出選擇,其中1表示基本上不可以,6表示完全可以。
在進行實驗之前,我們想瞭解您的一些基本信息。我們向您保證,您的這些信息將不會被洩露給任何個人與機構,而僅作為我們分析結果的參考。
填完問卷後，若出現抽獎活動為該問卷網頁的廣告而非本研究機構提供。請您填完後離開直接離開此網站即可。謝謝您的配合！請容我們再次為您說明，本問卷絕對不會要求個人個資信息，請您不要提供個人資訊給此網站的廣告。

請問您的年齡是: *

請問您的性別是: *

請問您的母語是: *

請問除母語外,您還會哪些語言: *

請問您的出生地是: *

請問您的長期生活地是: *

請問您的學歷是: *

(若適用)請問您所學的專業是:

1. 多大程度上,您認為(鹹)可以用來形容(味覺)? *

1(基本上不可以)	2	3	4	5	6(完全可以)

2. 多大程度上,您認為(泱泱)可以用來形容(味覺)? *

1(基本上不可以)	2	3	4	5	6(完全可以)

3. 多大程度上,您認為(郁郁)可以用來形容(聽覺)? *

1(基本上不可以)	2	3	4	5	6(完全可以)

4. 多大程度上,您認為(大)可以用來形容(嗅覺)? *

1(基本上不可以)	2	3	4	5	6(完全可以)

5. 多大程度上,您認為(閃閃)可以用來形容(味覺)? *

1(基本上不可以)	2	3	4	5	6(完全可以)

6. 多大程度上,您認為(鬆)可以用來形容(味覺)? *

1(基本上不可以)	2	3	4	5	6(完全可以)

7. 多大程度上,您認為(細)可以用來形容(觸覺)? *

1(基本上不可以)	2	3	4	5	6(完全可以)

8. 多大程度上,您認為(涼)可以用來形容(嗅覺)? *

1(基本上不可以)	2	3	4	5	6(完全可以)

9. 多大程度上,您認為(嫩)可以用來形容(觸覺)? *

1(基本上不可以)	2	3	4	5	6(完全可以)

10. 多大程度上,您認為(厚)可以用來形容(聽覺)? *

1(基本上不可以)	2	3	4	5	6(完全可以)

11. 多大程度上,您認為(盈盈)可以用來形容(視覺)? *

1(基本上不可以)	2	3	4	5	6(完全可以)

12. 多大程度上,您認為(鬆)可以用來形容(嗅覺)? *

1(基本上不可以)	2	3	4	5	6(完全可以)

13. 多大程度上,您認為(鳥)可以用來形容(味覺)? *

1(基本上不可以)	2	3	4	5	6(完全可以)

14. 多大程度上,您認為(鼓)可以用來形容(視覺)? *

1(基本上不可以)	2	3	4	5	6(完全可以)

15. 多大程度上,您認為(灼灼)可以用來形容(嗅覺)? *

1(基本上不可以)	2	3	4	5	6(完全可以)

16. 多大程度上,您認為(笨)可以用來形容(聽覺)? *

1(基本上不可以)	2	3	4	5	6(完全可以)

17. 多大程度上,您認為(熠熠)可以用來形容(視覺)? *

1(基本上不可以)	2	3	4	5	6(完全可以)

18. 多大程度上,您認為(蒼蒼)可以用來形容(視覺)? *

1(基本上不可以)	2	3	4	5	6(完全可以)

19. 多大程度上,您認為(正)可以用來形容(觸覺)? *

1(基本上不可以)	2	3	4	5	6(完全可以)

20. 多大程度上,您認為(暗)可以用來形容(聽覺)? *

1(基本上不可以)	2	3	4	5	6(完全可以)

21. 多大程度上,您認為(纖纖)可以用來形容(視覺)? *

1(基本上不可以)	2	3	4	5	6(完全可以)

22. 多大程度上,您認為(扁)可以用來形容(嗅覺)? *

1(基本上不可以)	2	3	4	5	6(完全可以)

23. 多大程度上,您認為(全)可以用來形容(聽覺)? *

1(基本上不可以)	2	3	4	5	6(完全可以)

24. 多大程度上,您認為(炎炎)可以用來形容(味覺)? *

1(基本上不可以)	2	3	4	5	6(完全可以)

25. 多大程度上,您認為(呆)可以用來形容(聽覺)? *

1(基本上不可以)	2	3	4	5	6(完全可以)

26. 多大程度上,您認為(滾滾)可以用來形容(觸覺)? *

1(基本上不可以)	2	3	4	5	6(完全可以)

27. 多大程度上,您認為(粉)可以用來形容(嗅覺)? *

1(基本上不可以)	2	3	4	5	6(完全可以)

28. 多大程度上,您認為(直)可以用來形容(聽覺)? *

1(基本上不可以)	2	3	4	5	6(完全可以)

29. 多大程度上,您認為(胖)可以用來形容(視覺)? *

1(基本上不可以)	2	3	4	5	6(完全可以)

30. 多大程度上,您認為(斜)可以用來形容(視覺)? *

1(基本上不可以)	2	3	4	5	6(完全可以)

31. 多大程度上,您認為(盈盈)可以用來形容(觸覺)? *

1(基本上不可以)	2	3	4	5	6(完全可以)

32. 多大程度上,您認為(木)可以用來形容(嗅覺)? *

1(基本上不可以)	2	3	4	5	6(完全可以)

33. 多大程度上,您認為(依依)可以用來形容(觸覺)? *

1(基本上不可以)	2	3	4	5	6(完全可以)

34. 多大程度上,您認為(紫)可以用來形容(嗅覺)? *

1(基本上不可以)	2	3	4	5	6(完全可以)

35. 多大程度上,您認為(濕)可以用來形容(聽覺)? *

1(基本上不可以)	2	3	4	5	6(完全可以)

36. 多大程度上,您認為(髒)可以用來形容(嗅覺)? *

1(基本上不可以)	2	3	4	5	6(完全可以)

37. 多大程度上,您認為(盈盈)可以用來形容(味覺)? *

1(基本上不可以)	2	3	4	5	6(完全可以)

38. 多大程度上,您認為(胖)可以用來形容(觸覺)? *

1(基本上不可以)	2	3	4	5	6(完全可以)

39. 多大程度上,您認為(緊)可以用來形容(觸覺)? *

1(基本上不可以)	2	3	4	5	6(完全可以)

40. 多大程度上,您認為(依依)可以用來形容(嗅覺)? *

1(基本上不可以)	2	3	4	5	6(完全可以)

41. 多大程度上,您認為(灰)可以用來形容(觸覺)? *

1(基本上不可以)	2	3	4	5	6(完全可以)

42. 多大程度上,您認為(亭亭)可以用來形容(聽覺)? *

1(基本上不可以)	2	3	4	5	6(完全可以)

43. 多大程度上,您認為(炯炯)可以用來形容(嗅覺)? *

1(基本上不可以)	2	3	4	5	6(完全可以)

44. 多大程度上,您認為(白)可以用來形容(聽覺)? *

1(基本上不可以)	2	3	4	5	6(完全可以)

45. 多大程度上,您認為(燥)可以用來形容(觸覺)? *

1(基本上不可以)	2	3	4	5	6(完全可以)

46. 多大程度上,您認為(壯)可以用來形容(觸覺)? *

1(基本上不可以)	2	3	4	5	6(完全可以)

47. 多大程度上,您認為(橫)可以用來形容(聽覺)? *

1(基本上不可以)	2	3	4	5	6(完全可以)

48. 多大程度上,您認為(胖)可以用來形容(聽覺)? *

1(基本上不可以)	2	3	4	5	6(完全可以)

49. 多大程度上,您認為(清)可以用來形容(觸覺)? *

1(基本上不可以)	2	3	4	5	6(完全可以)

50. 多大程度上,您認為(銳)可以用來形容(視覺)? *

1(基本上不可以)	2	3	4	5	6(完全可以)

51. 多大程度上,您認為(茫茫)可以用來形容(觸覺)? *

1(基本上不可以)	2	3	4	5	6(完全可以)

52. 多大程度上,您認為(青)可以用來形容(聽覺)? *

1(基本上不可以)	2	3	4	5	6(完全可以)

53. 多大程度上,您認為(鬆)可以用來形容(視覺)? *

1(基本上不可以)	2	3	4	5	6(完全可以)

54. 多大程度上,您認為(黃)可以用來形容(觸覺)? *

1(基本上不可以)	2	3	4	5	6(完全可以)

55. 多大程度上,您認為(薄)可以用來形容(味覺)? *

1(基本上不可以)	2	3	4	5	6(完全可以)

56. 多大程度上,您認為(泱泱)可以用來形容(視覺)? *

1(基本上不可以)	2	3	4	5	6(完全可以)

57. 多大程度上,您認為(炎炎)可以用來形容(嗅覺)? *

1(基本上不可以)	2	3	4	5	6(完全可以)

58. 多大程度上,您認為(烏)可以用來形容(視覺)? *

1(基本上不可以)	2	3	4	5	6(完全可以)

59. 多大程度上,您認為(甘)可以用來形容(聽覺)? *

1(基本上不可以)	2	3	4	5	6(完全可以)

60. 多大程度上,您認為(汪汪)可以用來形容(味覺)? *

1(基本上不可以)	2	3	4	5	6(完全可以)

提交

Powered By 问卷星
