# Supplementary material for: Mandarin Chinese modality exclusivity norms
Source: PLoS One. 2019 Feb 20;14(2):e0211336. doi: 10.1371/journal.pone.0211336 (PMC6382104; doi:10.1371/journal.pone.0211336)
Supplement: S4 File — (PDF) [file pone.0211336.s004.pdf]

感覺詞標註實驗(GROUP traditional)

說明: 人類的感覺大致可以分為5種,即視覺、聽覺、觸覺、味覺和嗅覺。因為有視覺,我們可以看到五彩繽紛的世界,因為有聽覺,我們能聽到各種各樣的聲音。而因為我們擁有觸覺,我們能感受到溫度的變化、物體材質的不同,偶爾我們的身體還能感受到疼痛等。與此同時,味覺能讓我們嚐到食物的不同味道,而嗅覺能讓我們聞到世界上各種不同的氣味。
在漢語中,有一批詞可以用來形容我們的感覺。在本次的實驗中,我們想邀請您來判斷,這些詞在多大程度上可以形容我們的這五種感覺。比如說,我們將給您如下的問題「多大程度上,您認為(清)可以用來形容視覺?」您將需要在1、2、3、4、5、6這六個選項中做出選擇,其中1表示基本上不可以,6表示完全可以。
在進行實驗之前,我們想瞭解您的一些基本信息。我們向您保證,您的這些信息將不會被洩露給任何個人與機構,而僅作為我們分析結果的參考。本實驗已通過香港理工大學的審核，問卷開始後表示您同意參與這項實驗，再次感謝您的協助!

请问您的年龄是/請問您的年齡是:

請問您的性別是:

請問您的母語是:

請問除母語外,您還會哪些語言:

請問您的出生地是:

請問您的長期生活地是:

請問您的學歷是:

(若適用)請問您所學的專業是:

1 多大的程度上,您認為(微弱)可以用來形容(嗅覺)? *

1(基本上不可以)	2	3	4	5	6(完全可以)"

2 多大的程度上,您認為(粗大)可以用來形容(視覺)? *

1(基本上不可以)	2	3	4	5	6(完全可以)"

3 多大的程度上,您認為(燦爛)可以用來形容(觸覺)? *

1(基本上不可以)	2	3	4	5	6(完全可以)"

4 多大的程度上,您認為(厚重)可以用來形容(味覺)? *

1(基本上不可以)	2	3	4	5	6(完全可以)"

5 多大的程度上,您認為(冷淡)可以用來形容(聽覺)? *

1(基本上不可以)	2	3	4	5	6(完全可以)"

6 多大的程度上,您認為(柔和)可以用來形容(嗅覺)? *

1(基本上不可以)	2	3	4	5	6(完全可以)"

7 多大的程度上,您認為(柔美)可以用來形容(視覺)? *

1(基本上不可以)	2	3	4	5	6(完全可以)"

8 多大的程度上,您認為(鬆軟)可以用來形容(聽覺)? *

1(基本上不可以)	2	3	4	5	6(完全可以)"

9 多大的程度上,您認為(微弱)可以用來形容(觸覺)? *

1(基本上不可以)	2	3	4	5	6(完全可以)"

10 多大的程度上,您認為(濃重)可以用來形容(味覺)? *

1(基本上不可以)	2	3	4	5	6(完全可以)"

11 多大的程度上,您認為(醇淨)可以用來形容(味覺)? *

1(基本上不可以)	2	3	4	5	6(完全可以)"

12 多大的程度上,您認為(厚重)可以用來形容(聽覺)? *

1(基本上不可以)	2	3	4	5	6(完全可以)"

13 多大的程度上,您認為(蒼涼)可以用來形容(嗅覺)? *

1(基本上不可以)	2	3	4	5	6(完全可以)"

14 多大的程度上,您認為(冷漠)可以用來形容(味覺)? *

1(基本上不可以)	2	3	4	5	6(完全可以)"

15 多大的程度上,您認為(濃烈)可以用來形容(視覺)? *

1(基本上不可以)	2	3	4	5	6(完全可以)"

16 多大的程度上,您認為(醇厚)可以用來形容(觸覺)? *

1(基本上不可以)	2	3	4	5	6(完全可以)"

17 多大的程度上,您認為(羸弱)可以用來形容(味覺)? *

1(基本上不可以)	2	3	4	5	6(完全可以)"

18 多大的程度上,您認為(平滑)可以用來形容(聽覺)? *

1(基本上不可以)	2	3	4	5	6(完全可以)"

19 多大的程度上,您認為(清甜)可以用來形容(嗅覺)? *

1(基本上不可以)	2	3	4	5	6(完全可以)"

20 多大的程度上,您認為(清冷)可以用來形容(觸覺)? *

1(基本上不可以)	2	3	4	5	6(完全可以)"

21 多大的程度上,您認為(鮮潤)可以用來形容(味覺)? *

1(基本上不可以)	2	3	4	5	6(完全可以)"

22 多大的程度上,您認為(溫和)可以用來形容(觸覺)? *

1(基本上不可以)	2	3	4	5	6(完全可以)"

23 多大的程度上,您認為(冷峻)可以用來形容(聽覺)? *

1(基本上不可以)	2	3	4	5	6(完全可以)"

24 多大的程度上,您認為(柔順)可以用來形容(視覺)? *

1(基本上不可以)	2	3	4	5	6(完全可以)"

25 多大的程度上,您認為(酸澀)可以用來形容(味覺)? *

1(基本上不可以)	2	3	4	5	6(完全可以)"

26 多大的程度上,您認為(圓潤)可以用來形容(觸覺)? *

1(基本上不可以)	2	3	4	5	6(完全可以)"

27 多大的程度上,您認為(淡然)可以用來形容(味覺)? *

1(基本上不可以)	2	3	4	5	6(完全可以)"

28 多大的程度上,您認為(沉重)可以用來形容(嗅覺)? *

1(基本上不可以)	2	3	4	5	6(完全可以)"

29 多大的程度上,您認為(酸澀)可以用來形容(嗅覺)? *

1(基本上不可以)	2	3	4	5	6(完全可以)"

30 多大的程度上,您認為(冷靜)可以用來形容(嗅覺)? *

1(基本上不可以)	2	3	4	5	6(完全可以)"

31 多大的程度上,您認為(柔細)可以用來形容(嗅覺)? *

1(基本上不可以)	2	3	4	5	6(完全可以)"

32 多大的程度上,您認為(平滑)可以用來形容(味覺)? *

1(基本上不可以)	2	3	4	5	6(完全可以)"

33 多大的程度上,您認為(清甜)可以用來形容(聽覺)? *

1(基本上不可以)	2	3	4	5	6(完全可以)"

34 多大的程度上,您認為(細滑)可以用來形容(視覺)? *

1(基本上不可以)	2	3	4	5	6(完全可以)"

35 多大的程度上,您認為(乾淨)可以用來形容(嗅覺)? *

1(基本上不可以)	2	3	4	5	6(完全可以)"

36 多大的程度上,您認為(羸弱)可以用來形容(觸覺)? *

1(基本上不可以)	2	3	4	5	6(完全可以)"

37 多大的程度上,您認為(濃郁)可以用來形容(聽覺)? *

1(基本上不可以)	2	3	4	5	6(完全可以)"

38 多大的程度上,您認為(清甜)可以用來形容(視覺)? *

1(基本上不可以)	2	3	4	5	6(完全可以)"

39 多大的程度上,您認為(甜潤)可以用來形容(味覺)? *

1(基本上不可以)	2	3	4	5	6(完全可以)"

40 多大的程度上,您認為(豐潤)可以用來形容(觸覺)? *

1(基本上不可以)	2	3	4	5	6(完全可以)"

41 多大的程度上,您認為(輕飄飄)可以用來形容(視覺)? *

1(基本上不可以)	2	3	4	5	6(完全可以)"

42 多大的程度上,您認為(燦爛)可以用來形容(嗅覺)? *

1(基本上不可以)	2	3	4	5	6(完全可以)"

43 多大的程度上,您認為(輕微)可以用來形容(聽覺)? *

1(基本上不可以)	2	3	4	5	6(完全可以)"

44 多大的程度上,您認為(清鮮)可以用來形容(視覺)? *

1(基本上不可以)	2	3	4	5	6(完全可以)"

45 多大的程度上,您認為(苦澀)可以用來形容(視覺)? *

1(基本上不可以)	2	3	4	5	6(完全可以)"

46 多大的程度上,您認為(沉重)可以用來形容(味覺)? *

1(基本上不可以)	2	3	4	5	6(完全可以)"

47 多大的程度上,您認為(痛苦)可以用來形容(觸覺)? *

1(基本上不可以)	2	3	4	5	6(完全可以)"

48 多大的程度上,您認為(輕盈)可以用來形容(味覺)? *

1(基本上不可以)	2	3	4	5	6(完全可以)"

49 多大的程度上,您認為(濃厚)可以用來形容(觸覺)? *

1(基本上不可以)	2	3	4	5	6(完全可以)"

50 多大的程度上,您認為(冷酷)可以用來形容(味覺)? *

1(基本上不可以)	2	3	4	5	6(完全可以)"

51 多大的程度上,您認為(柔美)可以用來形容(味覺)? *

1(基本上不可以)	2	3	4	5	6(完全可以)"

52 多大的程度上,您認為(豐潤)可以用來形容(聽覺)? *

1(基本上不可以)	2	3	4	5	6(完全可以)"

53 多大的程度上,您認為(硬繃繃)可以用來形容(視覺)? *

1(基本上不可以)	2	3	4	5	6(完全可以)"

54 多大的程度上,您認為(清甜)可以用來形容(味覺)? *

1(基本上不可以)	2	3	4	5	6(完全可以)"

55 多大的程度上,您認為(燦爛)可以用來形容(視覺)? *

1(基本上不可以)	2	3	4	5	6(完全可以)"

56 多大的程度上,您認為(冷酷)可以用來形容(視覺)? *

1(基本上不可以)	2	3	4	5	6(完全可以)"

57 多大的程度上,您認為(濃重)可以用來形容(視覺)? *

1(基本上不可以)	2	3	4	5	6(完全可以)"

58 多大的程度上,您認為(粗大)可以用來形容(聽覺)? *

1(基本上不可以)	2	3	4	5	6(完全可以)"

59 多大的程度上,您認為(青澀)可以用來形容(聽覺)? *

1(基本上不可以)	2	3	4	5	6(完全可以)"

60 多大的程度上,您認為(醇厚)可以用來形容(味覺)? *

1(基本上不可以)	2	3	4	5	6(完全可以)"

61 多大的程度上,您認為(苦楚)可以用來形容(嗅覺)? *

1(基本上不可以)	2	3	4	5	6(完全可以)"

62 多大的程度上,您認為(痛苦)可以用來形容(嗅覺)? *

1(基本上不可以)	2	3	4	5	6(完全可以)"

63 多大的程度上,您認為(淡然)可以用來形容(視覺)? *

1(基本上不可以)	2	3	4	5	6(完全可以)"

64 多大的程度上,您認為(軟綿綿)可以用來形容(嗅覺)? *

1(基本上不可以)	2	3	4	5	6(完全可以)"

65 多大的程度上,您認為(沉重)可以用來形容(視覺)? *

1(基本上不可以)	2	3	4	5	6(完全可以)"

66 多大的程度上,您認為(羸弱)可以用來形容(嗅覺)? *

1(基本上不可以)	2	3	4	5	6(完全可以)"

67 多大的程度上,您認為(燦爛)可以用來形容(味覺)? *

1(基本上不可以)	2	3	4	5	6(完全可以)"

68 多大的程度上,您認為(清鮮)可以用來形容(聽覺)? *

1(基本上不可以)	2	3	4	5	6(完全可以)"

69 多大的程度上,您認為(濕漉漉)可以用來形容(視覺)? *

1(基本上不可以)	2	3	4	5	6(完全可以)"

70 多大的程度上,您認為(嬌柔)可以用來形容(味覺)? *

1(基本上不可以)	2	3	4	5	6(完全可以)"

71 多大的程度上,您認為(濃郁)可以用來形容(味覺)? *

1(基本上不可以)	2	3	4	5	6(完全可以)"

72 多大的程度上,您認為(細膩)可以用來形容(嗅覺)? *

1(基本上不可以)	2	3	4	5	6(完全可以)"

73 多大的程度上,您認為(溫馨)可以用來形容(觸覺)? *

1(基本上不可以)	2	3	4	5	6(完全可以)"

74 多大的程度上,您認為(清冷)可以用來形容(嗅覺)? *

1(基本上不可以)	2	3	4	5	6(完全可以)"

75 多大的程度上,您認為(清冷)可以用來形容(視覺)? *

1(基本上不可以)	2	3	4	5	6(完全可以)"

76 多大的程度上,您認為(濃厚)可以用來形容(嗅覺)? *

1(基本上不可以)	2	3	4	5	6(完全可以)"

77 多大的程度上,您認為(虛弱)可以用來形容(視覺)? *

1(基本上不可以)	2	3	4	5	6(完全可以)"

78 多大的程度上,您認為(濃烈)可以用來形容(聽覺)? *

1(基本上不可以)	2	3	4	5	6(完全可以)"

79 多大的程度上,您認為(硬繃繃)可以用來形容(嗅覺)? *

1(基本上不可以)	2	3	4	5	6(完全可以)"

80 多大的程度上,您認為(柔順)可以用來形容(聽覺)? *

1(基本上不可以)	2	3	4	5	6(完全可以)"

81 多大的程度上,您認為(蒼涼)可以用來形容(味覺)? *

1(基本上不可以)	2	3	4	5	6(完全可以)"

82 多大的程度上,您認為(冷峻)可以用來形容(視覺)? *

1(基本上不可以)	2	3	4	5	6(完全可以)"

83 多大的程度上,您認為(微弱)可以用來形容(視覺)? *

1(基本上不可以)	2	3	4	5	6(完全可以)"

84 多大的程度上,您認為(熱辣)可以用來形容(觸覺)? *

1(基本上不可以)	2	3	4	5	6(完全可以)"

85 多大的程度上,您認為(溫和)可以用來形容(味覺)? *

1(基本上不可以)	2	3	4	5	6(完全可以)"

86 多大的程度上,您認為(清鮮)可以用來形容(觸覺)? *

1(基本上不可以)	2	3	4	5	6(完全可以)"

87 多大的程度上,您認為(豐潤)可以用來形容(味覺)? *

1(基本上不可以)	2	3	4	5	6(完全可以)"

88 多大的程度上,您認為(乾淨)可以用來形容(聽覺)? *

1(基本上不可以)	2	3	4	5	6(完全可以)"

89 多大的程度上,您認為(濃重)可以用來形容(觸覺)? *

1(基本上不可以)	2	3	4	5	6(完全可以)"

90 多大的程度上,您認為(冷然)可以用來形容(嗅覺)? *

1(基本上不可以)	2	3	4	5	6(完全可以)"

91 多大的程度上,您認為(平滑)可以用來形容(嗅覺)? *

1(基本上不可以)	2	3	4	5	6(完全可以)"

92 多大的程度上,您認為(蒼勁)可以用來形容(味覺)? *

1(基本上不可以)	2	3	4	5	6(完全可以)"

93 多大的程度上,您認為(醇淨)可以用來形容(嗅覺)? *

1(基本上不可以)	2	3	4	5	6(完全可以)"

94 多大的程度上,您認為(蒼涼)可以用來形容(聽覺)? *

1(基本上不可以)	2	3	4	5	6(完全可以)"

95 多大的程度上,您認為(冷漠)可以用來形容(嗅覺)? *

1(基本上不可以)	2	3	4	5	6(完全可以)"

96 多大的程度上,您認為(細滑)可以用來形容(聽覺)? *

1(基本上不可以)	2	3	4	5	6(完全可以)"

97 多大的程度上,您認為(冷靜)可以用來形容(視覺)? *

1(基本上不可以)	2	3	4	5	6(完全可以)"

98 多大的程度上,您認為(微弱)可以用來形容(味覺)? *

1(基本上不可以)	2	3	4	5	6(完全可以)"

99 多大的程度上,您認為(平淡)可以用來形容(味覺)? *

1(基本上不可以)	2	3	4	5	6(完全可以)"

100 多大的程度上,您認為(苦澀)可以用來形容(嗅覺)? *

1(基本上不可以)	2	3	4	5	6(完全可以)"

101 多大的程度上,您認為(粗大)可以用來形容(味覺)? *

1(基本上不可以)	2	3	4	5	6(完全可以)"

102 多大的程度上,您認為(苦楚)可以用來形容(味覺)? *

1(基本上不可以)	2	3	4	5	6(完全可以)"

103 多大的程度上,您認為(鮮潤)可以用來形容(嗅覺)? *

1(基本上不可以)	2	3	4	5	6(完全可以)"

104 多大的程度上,您認為(淡然)可以用來形容(觸覺)? *

1(基本上不可以)	2	3	4	5	6(完全可以)"

105 多大的程度上,您認為(溫和)可以用來形容(視覺)? *

1(基本上不可以)	2	3	4	5	6(完全可以)"

106 多大的程度上,您認為(虛弱)可以用來形容(味覺)? *

1(基本上不可以)	2	3	4	5	6(完全可以)"

107 多大的程度上,您認為(厚重)可以用來形容(嗅覺)? *

1(基本上不可以)	2	3	4	5	6(完全可以)"

108 多大的程度上,您認為(凝重)可以用來形容(聽覺)? *

1(基本上不可以)	2	3	4	5	6(完全可以)"

109 多大的程度上,您認為(柔和)可以用來形容(味覺)? *

1(基本上不可以)	2	3	4	5	6(完全可以)"

110 多大的程度上,您認為(輕鬆)可以用來形容(味覺)? *

1(基本上不可以)	2	3	4	5	6(完全可以)"

111 多大的程度上,您認為(輕微)可以用來形容(味覺)? *

1(基本上不可以)	2	3	4	5	6(完全可以)"

112 多大的程度上,您認為(粗大)可以用來形容(觸覺)? *

1(基本上不可以)	2	3	4	5	6(完全可以)"

113 多大的程度上,您認為(冷淡)可以用來形容(味覺)? *

1(基本上不可以)	2	3	4	5	6(完全可以)"

114 多大的程度上,您認為(輕盈)可以用來形容(嗅覺)? *

1(基本上不可以)	2	3	4	5	6(完全可以)"

115 多大的程度上,您認為(痛苦)可以用來形容(聽覺)? *

1(基本上不可以)	2	3	4	5	6(完全可以)"

116 多大的程度上,您認為(濃郁)可以用來形容(視覺)? *

1(基本上不可以)	2	3	4	5	6(完全可以)"

117 多大的程度上,您認為(痛苦)可以用來形容(味覺)? *

1(基本上不可以)	2	3	4	5	6(完全可以)"

118 多大的程度上,您認為(濃厚)可以用來形容(味覺)? *

1(基本上不可以)	2	3	4	5	6(完全可以)"

119 多大的程度上,您認為(冷靜)可以用來形容(味覺)? *

1(基本上不可以)	2	3	4	5	6(完全可以)"

120 多大的程度上,您認為(細膩)可以用來形容(聽覺)? *

1(基本上不可以)	2	3	4	5	6(完全可以)"

121 多大的程度上,您認為(冷峻)可以用來形容(嗅覺)? *

1(基本上不可以)	2	3	4	5	6(完全可以)"

122 多大的程度上,您認為(淡然)可以用來形容(聽覺)? *

1(基本上不可以)	2	3	4	5	6(完全可以)"

123 多大的程度上,您認為(冷酷)可以用來形容(嗅覺)? *

1(基本上不可以)	2	3	4	5	6(完全可以)"

124 多大的程度上,您認為(濃烈)可以用來形容(觸覺)? *

1(基本上不可以)	2	3	4	5	6(完全可以)"

125 多大的程度上,您認為(細滑)可以用來形容(觸覺)? *

1(基本上不可以)	2	3	4	5	6(完全可以)"

126 多大的程度上,您認為(輕微)可以用來形容(視覺)? *

1(基本上不可以)	2	3	4	5	6(完全可以)"

127 多大的程度上,您認為(柔細)可以用來形容(味覺)? *

1(基本上不可以)	2	3	4	5	6(完全可以)"

128 多大的程度上,您認為(冷酷)可以用來形容(觸覺)? *

1(基本上不可以)	2	3	4	5	6(完全可以)"

129 多大的程度上,您認為(嬌柔)可以用來形容(聽覺)? *

1(基本上不可以)	2	3	4	5	6(完全可以)"

130 多大的程度上,您認為(苦楚)可以用來形容(視覺)? *

1(基本上不可以)	2	3	4	5	6(完全可以)"

131 多大的程度上,您認為(柔順)可以用來形容(味覺)? *

1(基本上不可以)	2	3	4	5	6(完全可以)"

132 多大的程度上,您認為(鬆軟)可以用來形容(視覺)? *

1(基本上不可以)	2	3	4	5	6(完全可以)"

133 多大的程度上,您認為(酸澀)可以用來形容(視覺)? *

1(基本上不可以)	2	3	4	5	6(完全可以)"

134 多大的程度上,您認為(濃重)可以用來形容(嗅覺)? *

1(基本上不可以)	2	3	4	5	6(完全可以)"

135 多大的程度上,您認為(濕漉漉)可以用來形容(味覺)? *

1(基本上不可以)	2	3	4	5	6(完全可以)"

136 多大的程度上,您認為(火辣)可以用來形容(視覺)? *

1(基本上不可以)	2	3	4	5	6(完全可以)"

137 多大的程度上,您認為(淡然)可以用來形容(嗅覺)? *

1(基本上不可以)	2	3	4	5	6(完全可以)"

138 多大的程度上,您認為(溫馨)可以用來形容(聽覺)? *

1(基本上不可以)	2	3	4	5	6(完全可以)"

139 多大的程度上,您認為(凝重)可以用來形容(觸覺)? *

1(基本上不可以)	2	3	4	5	6(完全可以)"

140 多大的程度上,您認為(火辣)可以用來形容(味覺)? *

1(基本上不可以)	2	3	4	5	6(完全可以)"

141 多大的程度上,您認為(柔美)可以用來形容(聽覺)? *

1(基本上不可以)	2	3	4	5	6(完全可以)"

142 多大的程度上,您認為(圓潤)可以用來形容(視覺)? *

1(基本上不可以)	2	3	4	5	6(完全可以)"

143 多大的程度上,您認為(甜潤)可以用來形容(觸覺)? *

1(基本上不可以)	2	3	4	5	6(完全可以)"

144 多大的程度上,您認為(嬌柔)可以用來形容(觸覺)? *

1(基本上不可以)	2	3	4	5	6(完全可以)"

145 多大的程度上,您認為(輕盈)可以用來形容(視覺)? *

1(基本上不可以)	2	3	4	5	6(完全可以)"

146 多大的程度上,您認為(溫和)可以用來形容(嗅覺)? *

1(基本上不可以)	2	3	4	5	6(完全可以)"

147 多大的程度上,您認為(醇淨)可以用來形容(觸覺)? *

1(基本上不可以)	2	3	4	5	6(完全可以)"

148 多大的程度上,您認為(虛弱)可以用來形容(觸覺)? *

1(基本上不可以)	2	3	4	5	6(完全可以)"

149 多大的程度上,您認為(清淡)可以用來形容(觸覺)? *

1(基本上不可以)	2	3	4	5	6(完全可以)"

150 多大的程度上,您認為(粗壯)可以用來形容(味覺)? *

1(基本上不可以)	2	3	4	5	6(完全可以)"

151 多大的程度上,您認為(淒涼)可以用來形容(觸覺)? *

1(基本上不可以)	2	3	4	5	6(完全可以)"

152 多大的程度上,您認為(醇淨)可以用來形容(視覺)? *

1(基本上不可以)	2	3	4	5	6(完全可以)"

153 多大的程度上,您認為(熱辣)可以用來形容(聽覺)? *

1(基本上不可以)	2	3	4	5	6(完全可以)"

154 多大的程度上,您認為(沉重)可以用來形容(聽覺)? *

1(基本上不可以)	2	3	4	5	6(完全可以)"

155 多大的程度上,您認為(青澀)可以用來形容(味覺)? *

1(基本上不可以)	2	3	4	5	6(完全可以)"

156 多大的程度上,您認為(硬繃繃)可以用來形容(觸覺)? *

1(基本上不可以)	2	3	4	5	6(完全可以)"

157 多大的程度上,您認為(濃郁)可以用來形容(嗅覺)? *

1(基本上不可以)	2	3	4	5	6(完全可以)"

158 多大的程度上,您認為(柔和)可以用來形容(聽覺)? *

1(基本上不可以)	2	3	4	5	6(完全可以)"

159 多大的程度上,您認為(冷然)可以用來形容(味覺)? *

1(基本上不可以)	2	3	4	5	6(完全可以)"

160 多大的程度上,您認為(鮮潤)可以用來形容(觸覺)? *

1(基本上不可以)	2	3	4	5	6(完全可以)"

161 多大的程度上,您認為(蒼勁)可以用來形容(視覺)? *

1(基本上不可以)	2	3	4	5	6(完全可以)"

162 多大的程度上,您認為(鮮潤)可以用來形容(視覺)? *

1(基本上不可以)	2	3	4	5	6(完全可以)"

163 多大的程度上,您認為(細滑)可以用來形容(嗅覺)? *

1(基本上不可以)	2	3	4	5	6(完全可以)"

164 多大的程度上,您認為(平淡)可以用來形容(聽覺)? *

1(基本上不可以)	2	3	4	5	6(完全可以)"

165 多大的程度上,您認為(平滑)可以用來形容(視覺)? *

1(基本上不可以)	2	3	4	5	6(完全可以)"

166 多大的程度上,您認為(柔細)可以用來形容(觸覺)? *

1(基本上不可以)	2	3	4	5	6(完全可以)"

167 多大的程度上,您認為(輕飄飄)可以用來形容(味覺)? *

1(基本上不可以)	2	3	4	5	6(完全可以)"

168 多大的程度上,您認為(輕微)可以用來形容(嗅覺)? *

1(基本上不可以)	2	3	4	5	6(完全可以)"

169 多大的程度上,您認為(冷峻)可以用來形容(觸覺)? *

1(基本上不可以)	2	3	4	5	6(完全可以)"

170 多大的程度上,您認為(細膩)可以用來形容(視覺)? *

1(基本上不可以)	2	3	4	5	6(完全可以)"

171 多大的程度上,您認為(冷靜)可以用來形容(觸覺)? *

1(基本上不可以)	2	3	4	5	6(完全可以)"

172 多大的程度上,您認為(溫和)可以用來形容(聽覺)? *

1(基本上不可以)	2	3	4	5	6(完全可以)"

173 多大的程度上,您認為(輕飄飄)可以用來形容(聽覺)? *

1(基本上不可以)	2	3	4	5	6(完全可以)"

174 多大的程度上,您認為(嬌柔)可以用來形容(嗅覺)? *

1(基本上不可以)	2	3	4	5	6(完全可以)"

175 多大的程度上,您認為(微弱)可以用來形容(聽覺)? *

1(基本上不可以)	2	3	4	5	6(完全可以)"

176 多大的程度上,您認為(蒼勁)可以用來形容(聽覺)? *

1(基本上不可以)	2	3	4	5	6(完全可以)"

177 多大的程度上,您認為(凝重)可以用來形容(嗅覺)? *

1(基本上不可以)	2	3	4	5	6(完全可以)"

178 多大的程度上,您認為(淒涼)可以用來形容(嗅覺)? *

1(基本上不可以)	2	3	4	5	6(完全可以)"

179 多大的程度上,您認為(柔順)可以用來形容(觸覺)? *

1(基本上不可以)	2	3	4	5	6(完全可以)"

180 多大的程度上,您認為(濃烈)可以用來形容(味覺)? *

1(基本上不可以)	2	3	4	5	6(完全可以)"

181 多大的程度上,您認為(平淡)可以用來形容(嗅覺)? *

1(基本上不可以)	2	3	4	5	6(完全可以)"

182 多大的程度上,您認為(鮮潤)可以用來形容(聽覺)? *

1(基本上不可以)	2	3	4	5	6(完全可以)"

183 多大的程度上,您認為(粗壯)可以用來形容(嗅覺)? *

1(基本上不可以)	2	3	4	5	6(完全可以)"

184 多大的程度上,您認為(清冷)可以用來形容(味覺)? *

1(基本上不可以)	2	3	4	5	6(完全可以)"

185 多大的程度上,您認為(清鮮)可以用來形容(嗅覺)? *

1(基本上不可以)	2	3	4	5	6(完全可以)"

186 多大的程度上,您認為(圓潤)可以用來形容(嗅覺)? *

1(基本上不可以)	2	3	4	5	6(完全可以)"

187 多大的程度上,您認為(輕鬆)可以用來形容(聽覺)? *

1(基本上不可以)	2	3	4	5	6(完全可以)"

188 多大的程度上,您認為(清淡)可以用來形容(視覺)? *

1(基本上不可以)	2	3	4	5	6(完全可以)"

189 多大的程度上,您認為(冷淡)可以用來形容(嗅覺)? *

1(基本上不可以)	2	3	4	5	6(完全可以)"

190 多大的程度上,您認為(冷漠)可以用來形容(聽覺)? *

1(基本上不可以)	2	3	4	5	6(完全可以)"

191 多大的程度上,您認為(豐潤)可以用來形容(嗅覺)? *

1(基本上不可以)	2	3	4	5	6(完全可以)"

192 多大的程度上,您認為(濃重)可以用來形容(聽覺)? *

1(基本上不可以)	2	3	4	5	6(完全可以)"

193 多大的程度上,您認為(輕飄飄)可以用來形容(觸覺)? *

1(基本上不可以)	2	3	4	5	6(完全可以)"

194 多大的程度上,您認為(輕鬆)可以用來形容(嗅覺)? *

1(基本上不可以)	2	3	4	5	6(完全可以)"

195 多大的程度上,您認為(酸澀)可以用來形容(聽覺)? *

1(基本上不可以)	2	3	4	5	6(完全可以)"

196 多大的程度上,您認為(苦楚)可以用來形容(觸覺)? *

1(基本上不可以)	2	3	4	5	6(完全可以)"

197 多大的程度上,您認為(嬌柔)可以用來形容(視覺)? *

1(基本上不可以)	2	3	4	5	6(完全可以)"

198 多大的程度上,您認為(冷淡)可以用來形容(觸覺)? *

1(基本上不可以)	2	3	4	5	6(完全可以)"

199 多大的程度上,您認為(濕漉漉)可以用來形容(觸覺)? *

1(基本上不可以)	2	3	4	5	6(完全可以)"

200 多大的程度上,您認為(醇厚)可以用來形容(視覺)? *

1(基本上不可以)	2	3	4	5	6(完全可以)"

201 多大的程度上,您認為(輕盈)可以用來形容(聽覺)? *

1(基本上不可以)	2	3	4	5	6(完全可以)"

202 多大的程度上,您認為(鬆軟)可以用來形容(味覺)? *

1(基本上不可以)	2	3	4	5	6(完全可以)"

203 多大的程度上,您認為(柔細)可以用來形容(聽覺)? *

1(基本上不可以)	2	3	4	5	6(完全可以)"

204 多大的程度上,您認為(細膩)可以用來形容(味覺)? *

1(基本上不可以)	2	3	4	5	6(完全可以)"

205 多大的程度上,您認為(濃郁)可以用來形容(觸覺)? *

1(基本上不可以)	2	3	4	5	6(完全可以)"

206 多大的程度上,您認為(輕微)可以用來形容(觸覺)? *

1(基本上不可以)	2	3	4	5	6(完全可以)"

207 多大的程度上,您認為(清淡)可以用來形容(嗅覺)? *

1(基本上不可以)	2	3	4	5	6(完全可以)"

208 多大的程度上,您認為(虛弱)可以用來形容(聽覺)? *

1(基本上不可以)	2	3	4	5	6(完全可以)"

209 多大的程度上,您認為(熱辣)可以用來形容(味覺)? *

1(基本上不可以)	2	3	4	5	6(完全可以)"

210 多大的程度上,您認為(細滑)可以用來形容(味覺)? *

1(基本上不可以)	2	3	4	5	6(完全可以)"

211 多大的程度上,您認為(羸弱)可以用來形容(聽覺)? *

1(基本上不可以)	2	3	4	5	6(完全可以)"

212 多大的程度上,您認為(青澀)可以用來形容(視覺)? *

1(基本上不可以)	2	3	4	5	6(完全可以)"

213 多大的程度上,您認為(平滑)可以用來形容(觸覺)? *

1(基本上不可以)	2	3	4	5	6(完全可以)"

214 多大的程度上,您認為(柔細)可以用來形容(視覺)? *

1(基本上不可以)	2	3	4	5	6(完全可以)"

215 多大的程度上,您認為(清淡)可以用來形容(味覺)? *

1(基本上不可以)	2	3	4	5	6(完全可以)"

216 多大的程度上,您認為(清甜)可以用來形容(觸覺)? *

1(基本上不可以)	2	3	4	5	6(完全可以)"

217 多大的程度上,您認為(蒼勁)可以用來形容(觸覺)? *

1(基本上不可以)	2	3	4	5	6(完全可以)"

218 多大的程度上,您認為(柔美)可以用來形容(嗅覺)? *

1(基本上不可以)	2	3	4	5	6(完全可以)"

219 多大的程度上,您認為(清脆)可以用來形容(視覺)? *

1(基本上不可以)	2	3	4	5	6(完全可以)"

220 多大的程度上,您認為(蒼涼)可以用來形容(觸覺)? *

1(基本上不可以)	2	3	4	5	6(完全可以)"

221 多大的程度上,您認為(圓潤)可以用來形容(味覺)? *

1(基本上不可以)	2	3	4	5	6(完全可以)"

222 多大的程度上,您認為(冷淡)可以用來形容(視覺)? *

1(基本上不可以)	2	3	4	5	6(完全可以)"

223 多大的程度上,您認為(乾淨)可以用來形容(視覺)? *

1(基本上不可以)	2	3	4	5	6(完全可以)"

224 多大的程度上,您認為(清冷)可以用來形容(聽覺)? *

1(基本上不可以)	2	3	4	5	6(完全可以)"

225 多大的程度上,您認為(豐潤)可以用來形容(視覺)? *

1(基本上不可以)	2	3	4	5	6(完全可以)"

226 多大的程度上,您認為(清脆)可以用來形容(嗅覺)? *

1(基本上不可以)	2	3	4	5	6(完全可以)"

227 多大的程度上,您認為(冷然)可以用來形容(聽覺)? *

1(基本上不可以)	2	3	4	5	6(完全可以)"

228 多大的程度上,您認為(柔美)可以用來形容(觸覺)? *

1(基本上不可以)	2	3	4	5	6(完全可以)"

229 多大的程度上,您認為(厚重)可以用來形容(視覺)? *

1(基本上不可以)	2	3	4	5	6(完全可以)"

230 多大的程度上,您認為(濃烈)可以用來形容(嗅覺)? *

1(基本上不可以)	2	3	4	5	6(完全可以)"

231 多大的程度上,您認為(火辣)可以用來形容(嗅覺)? *

1(基本上不可以)	2	3	4	5	6(完全可以)"

232 多大的程度上,您認為(清脆)可以用來形容(聽覺)? *

1(基本上不可以)	2	3	4	5	6(完全可以)"

233 多大的程度上,您認為(醇厚)可以用來形容(嗅覺)? *

1(基本上不可以)	2	3	4	5	6(完全可以)"

234 多大的程度上,您認為(乾淨)可以用來形容(觸覺)? *

1(基本上不可以)	2	3	4	5	6(完全可以)"

235 多大的程度上,您認為(凝重)可以用來形容(視覺)? *

1(基本上不可以)	2	3	4	5	6(完全可以)"

236 多大的程度上,您認為(青澀)可以用來形容(嗅覺)? *

1(基本上不可以)	2	3	4	5	6(完全可以)"

237 多大的程度上,您認為(圓潤)可以用來形容(聽覺)? *

1(基本上不可以)	2	3	4	5	6(完全可以)"

238 多大的程度上,您認為(鬆軟)可以用來形容(嗅覺)? *

1(基本上不可以)	2	3	4	5	6(完全可以)"

239 多大的程度上,您認為(熱辣)可以用來形容(嗅覺)? *

1(基本上不可以)	2	3	4	5	6(完全可以)"

240 多大的程度上,您認為(苦楚)可以用來形容(聽覺)? *

1(基本上不可以)	2	3	4	5	6(完全可以)"

241 多大的程度上,您認為(冷漠)可以用來形容(觸覺)? *

1(基本上不可以)	2	3	4	5	6(完全可以)"

242 多大的程度上,您認為(冷然)可以用來形容(視覺)? *

1(基本上不可以)	2	3	4	5	6(完全可以)"

243 多大的程度上,您認為(清鮮)可以用來形容(味覺)? *

1(基本上不可以)	2	3	4	5	6(完全可以)"

244 多大的程度上,您認為(軟綿綿)可以用來形容(觸覺)? *

1(基本上不可以)	2	3	4	5	6(完全可以)"

245 多大的程度上,您認為(痛苦)可以用來形容(視覺)? *

1(基本上不可以)	2	3	4	5	6(完全可以)"

246 多大的程度上,您認為(溫馨)可以用來形容(視覺)? *

1(基本上不可以)	2	3	4	5	6(完全可以)"

247 多大的程度上,您認為(溫馨)可以用來形容(嗅覺)? *

1(基本上不可以)	2	3	4	5	6(完全可以)"

248 多大的程度上,您認為(冷漠)可以用來形容(視覺)? *

1(基本上不可以)	2	3	4	5	6(完全可以)"

249 多大的程度上,您認為(酸澀)可以用來形容(觸覺)? *

1(基本上不可以)	2	3	4	5	6(完全可以)"

250 多大的程度上,您認為(平淡)可以用來形容(觸覺)? *

1(基本上不可以)	2	3	4	5	6(完全可以)"

251 多大的程度上,您認為(淒涼)可以用來形容(視覺)? *

1(基本上不可以)	2	3	4	5	6(完全可以)"

252 多大的程度上,您認為(苦澀)可以用來形容(觸覺)? *

1(基本上不可以)	2	3	4	5	6(完全可以)"

253 多大的程度上,您認為(乾淨)可以用來形容(味覺)? *

1(基本上不可以)	2	3	4	5	6(完全可以)"

254 多大的程度上,您認為(平淡)可以用來形容(視覺)? *

1(基本上不可以)	2	3	4	5	6(完全可以)"

255 多大的程度上,您認為(鬆軟)可以用來形容(觸覺)? *

1(基本上不可以)	2	3	4	5	6(完全可以)"

256 多大的程度上,您認為(甜潤)可以用來形容(嗅覺)? *

1(基本上不可以)	2	3	4	5	6(完全可以)"

257 多大的程度上,您認為(柔和)可以用來形容(視覺)? *

1(基本上不可以)	2	3	4	5	6(完全可以)"

258 多大的程度上,您認為(細膩)可以用來形容(觸覺)? *

1(基本上不可以)	2	3	4	5	6(完全可以)"

259 多大的程度上,您認為(濕漉漉)可以用來形容(嗅覺)? *

1(基本上不可以)	2	3	4	5	6(完全可以)"

260 多大的程度上,您認為(輕盈)可以用來形容(觸覺)? *

1(基本上不可以)	2	3	4	5	6(完全可以)"

261 多大的程度上,您認為(柔順)可以用來形容(嗅覺)? *

1(基本上不可以)	2	3	4	5	6(完全可以)"

262 多大的程度上,您認為(軟綿綿)可以用來形容(視覺)? *

1(基本上不可以)	2	3	4	5	6(完全可以)"

263 多大的程度上,您認為(甜潤)可以用來形容(聽覺)? *

1(基本上不可以)	2	3	4	5	6(完全可以)"

264 多大的程度上,您認為(輕飄飄)可以用來形容(嗅覺)? *

1(基本上不可以)	2	3	4	5	6(完全可以)"

265 多大的程度上,您認為(清脆)可以用來形容(味覺)? *

1(基本上不可以)	2	3	4	5	6(完全可以)"

266 多大的程度上,您認為(軟綿綿)可以用來形容(聽覺)? *

1(基本上不可以)	2	3	4	5	6(完全可以)"

267 多大的程度上,您認為(硬繃繃)可以用來形容(聽覺)? *

1(基本上不可以)	2	3	4	5	6(完全可以)"

268 多大的程度上,您認為(淒涼)可以用來形容(味覺)? *

1(基本上不可以)	2	3	4	5	6(完全可以)"

269 多大的程度上,您認為(冷靜)可以用來形容(聽覺)? *

1(基本上不可以)	2	3	4	5	6(完全可以)"

270 多大的程度上,您認為(濃厚)可以用來形容(視覺)? *

1(基本上不可以)	2	3	4	5	6(完全可以)"

271 多大的程度上,您認為(燦爛)可以用來形容(聽覺)? *

1(基本上不可以)	2	3	4	5	6(完全可以)"

272 多大的程度上,您認為(蒼勁)可以用來形容(嗅覺)? *

1(基本上不可以)	2	3	4	5	6(完全可以)"

273 多大的程度上,您認為(濃厚)可以用來形容(聽覺)? *

1(基本上不可以)	2	3	4	5	6(完全可以)"

274 多大的程度上,您認為(淒涼)可以用來形容(聽覺)? *

1(基本上不可以)	2	3	4	5	6(完全可以)"

275 多大的程度上,您認為(火辣)可以用來形容(觸覺)? *

1(基本上不可以)	2	3	4	5	6(完全可以)"

276 多大的程度上,您認為(冷峻)可以用來形容(味覺)? *

1(基本上不可以)	2	3	4	5	6(完全可以)"

277 多大的程度上,您認為(厚重)可以用來形容(觸覺)? *

1(基本上不可以)	2	3	4	5	6(完全可以)"

278 多大的程度上,您認為(硬繃繃)可以用來形容(味覺)? *

1(基本上不可以)	2	3	4	5	6(完全可以)"

279 多大的程度上,您認為(粗大)可以用來形容(嗅覺)? *

1(基本上不可以)	2	3	4	5	6(完全可以)"

280 多大的程度上,您認為(冷酷)可以用來形容(聽覺)? *

1(基本上不可以)	2	3	4	5	6(完全可以)"

281 多大的程度上,您認為(凝重)可以用來形容(味覺)? *

1(基本上不可以)	2	3	4	5	6(完全可以)"

282 多大的程度上,您認為(輕鬆)可以用來形容(觸覺)? *

1(基本上不可以)	2	3	4	5	6(完全可以)"

283 多大的程度上,您認為(冷然)可以用來形容(觸覺)? *

1(基本上不可以)	2	3	4	5	6(完全可以)"

284 多大的程度上,您認為(清淡)可以用來形容(聽覺)? *

1(基本上不可以)	2	3	4	5	6(完全可以)"

285 多大的程度上,您認為(粗壯)可以用來形容(聽覺)? *

1(基本上不可以)	2	3	4	5	6(完全可以)"

286 多大的程度上,您認為(甜潤)可以用來形容(視覺)? *

1(基本上不可以)	2	3	4	5	6(完全可以)"

287 多大的程度上,您認為(清脆)可以用來形容(觸覺)? *

1(基本上不可以)	2	3	4	5	6(完全可以)"

288 多大的程度上,您認為(溫馨)可以用來形容(味覺)? *

1(基本上不可以)	2	3	4	5	6(完全可以)"

289 多大的程度上,您認為(粗壯)可以用來形容(觸覺)? *

1(基本上不可以)	2	3	4	5	6(完全可以)"

290 多大的程度上,您認為(沉重)可以用來形容(觸覺)? *

1(基本上不可以)	2	3	4	5	6(完全可以)"

291 多大的程度上,您認為(苦澀)可以用來形容(味覺)? *

1(基本上不可以)	2	3	4	5	6(完全可以)"

292 多大的程度上,您認為(粗壯)可以用來形容(視覺)? *

1(基本上不可以)	2	3	4	5	6(完全可以)"

293 多大的程度上,您認為(羸弱)可以用來形容(視覺)? *

1(基本上不可以)	2	3	4	5	6(完全可以)"

294 多大的程度上,您認為(醇淨)可以用來形容(聽覺)? *

1(基本上不可以)	2	3	4	5	6(完全可以)"

295 多大的程度上,您認為(苦澀)可以用來形容(聽覺)? *

1(基本上不可以)	2	3	4	5	6(完全可以)"

296 多大的程度上,您認為(濕漉漉)可以用來形容(聽覺)? *

1(基本上不可以)	2	3	4	5	6(完全可以)"

297 多大的程度上,您認為(火辣)可以用來形容(聽覺)? *

1(基本上不可以)	2	3	4	5	6(完全可以)"

298 多大的程度上,您認為(蒼涼)可以用來形容(視覺)? *

1(基本上不可以)	2	3	4	5	6(完全可以)"

299 多大的程度上,您認為(醇厚)可以用來形容(聽覺)? *

1(基本上不可以)	2	3	4	5	6(完全可以)"

300 多大的程度上,您認為(軟綿綿)可以用來形容(味覺)? *

1(基本上不可以)	2	3	4	5	6(完全可以)"

301 多大的程度上,您認為(柔和)可以用來形容(觸覺)? *

1(基本上不可以)	2	3	4	5	6(完全可以)"

302 多大的程度上,您認為(虛弱)可以用來形容(嗅覺)? *

1(基本上不可以)	2	3	4	5	6(完全可以)"

303 多大的程度上,您認為(青澀)可以用來形容(觸覺)? *

1(基本上不可以)	2	3	4	5	6(完全可以)"

304 多大的程度上,您認為(熱辣)可以用來形容(視覺)? *

1(基本上不可以)	2	3	4	5	6(完全可以)"

305 多大的程度上,您認為(輕鬆)可以用來形容(視覺)? *

1(基本上不可以)	2	3	4	5	6(完全可以)"

306 多大的程度上,您認為(粗壯)可以用來形容(視覺)? *

1(基本上不可以)	2	3	4	5	6(完全可以)"

提交

Powered By 问卷星
